# Partitioning and Translocation of Dry Matter and Nitrogen During Grain Filling in Spring Barley Varieties and Their Roles in Determining Malting Quality

**DOI:** 10.3389/fpls.2021.722871

**Published:** 2021-08-23

**Authors:** Gero Barmeier, Yuncai Hu, Urs Schmidhalter

**Affiliations:** Department of Plant Sciences, Technical University of Munich, Freising, Germany

**Keywords:** dry matter, genotype, malting quality, nitrogen, partitioning, remobilization, spring barley

## Abstract

To meet the strict requirements for the malting quality of both grain size and protein content for malting barley, a better understanding of the partitioning and remobilization of dry matter (DM) and nitrogen (N) from individual vegetative organs during grain filling may contribute to adjusting a balance in both quality parameters to satisfy the malting criteria of the brewing industry. A 2-year experiment that included 23 spring malting barley varieties was carried out to determine the DM and N partitioning in different organs at anthesis and maturity and to estimate their remobilization to grains. In contrast to the genetic variation of the 23 barley varieties, year effect was the most important single factor influencing the DM and N accumulation at pre-anthesis, and the DM and N translocation from their reserves at pre-anthesis. Post-anthesis assimilates accounted for 71–94% of the total grain yield among the barley varieties in 2014 and 53–81% in 2015. In contrast, the N reserved in vegetative tissues at anthesis contributed to barley grain N from 67% in the variety Union to 91% in the variety Marthe in 2014, and 71% in the variety Grace to 97% in the variety Shakira in 2015. The results concluded that photosynthetically derived assimilates at post-anthesis played an important role in determining grain size, whereas N reserves at pre-anthesis and N remobilization at post-anthesis probably determined the grain protein content of the malting barley. To achieve a high quality of malting barley grains in both grain size and protein content simultaneously, balancing photosynthetic assimilates at post-anthesis and N reserves at pre-anthesis and N remobilization should be considered as strategies for the combination of the selection of spring malting barley varieties together with agronomic N management.

## Introduction

Barley is unique among crop plants and is the fourth most important crop globally among cereals after maize, rice, and wheat (Newton et al., 2011). Barley grain, in the form of malt, is a nutritional source for yeast, which is very important for the brewing industry. In addition to achieving high yield, both grain protein content and size that are specific quality criteria for malting barley must be met to maximize the efficiency of the malting process and the quality of the products. The requirements for grain protein content and size range from 9.5 to 12.5% and >2.38 mm in North America (Shrestha and Lindsey, 2019), while the grain protein content is from 10 to 12%, and the requirement for the grain size is >2.5 mm (>70%) and <2.2 mm (<5%) in Australia (Fox et al., 2003). In France and Germany, the malting barley grain protein content must be in the range of 9.5 to 11.5% of the dry weight, and the retention fraction (proportion of grains larger than 2.5 mm) must be >90% (Incograin, 2014; Bundessortenamt, 2016; Beillouin et al., 2018). If they do not meet these requirements, the grains can be downgraded to feed barley, resulting in a much lower price paid to farmers (Incograin, 2014; Bundessortenamt, 2016). The grain quality of malting barley is very variable because of genotypic and environmental effects (Carreck and Christian, 1991; Atanassov et al., 1999; Savin et al., 2006; Vahamidis et al., 2021), making it challenging to meet quality standards. Since variation in annual weather conditions leads to different influences on grain retention fraction and grain protein content, the same varieties may show a large variation in the annual quality parameters (Savin et al., 2006; Hu et al., 2021). Therefore, to meet the strict requirements for both quality parameters, it is necessary to understand the partitioning and remobilization of dry matter (DM) and nitrogen (N) from vegetative organs of malting barley at pre-anthesis and post-anthesis photosynthetic assimilation and N uptake, which may regulate the balance between the grain size and protein content in different varieties of malting barley.

Post-anthesis stages of barley play a crucial role in balancing grain protein content and grain size (Borras et al., 2004). During grain filling of spring barley, the supply to grains may originate from both post-anthesis current photosynthetic assimilation or N uptake and remobilization of their reserves at pre-anthesis in the vegetative parts of the plant, such as leaves, stems, sheaths, and chaff (Austin et al., 1980; van Sanford and Mackown, 1987; Borras et al., 2004). Grain DM is sink-limited (Borras et al., 2004) and mostly acquired by photosynthesis during grain filling, while DM remobilization from the reserves in vegetative organs to grains during grain filling only reaches approximately 10% of barley grain yield (Austin et al., 1980; Przulj and Momcilovic, 2001a,b; Dordas, 2012). In contrast, pre-anthesis-accumulated N seems to be the predominant source of N during grain filling (van Sanford and Mackown, 1987). The amount of N at anthesis in the aboveground parts of cereal crops can be as high as >90% of the total plant N at maturity (Clarke et al., 1990; Heitholt et al., 1990). Although studies have reported N remobilization during grain filling in barley, this has invariably been at a coarse level, and included remobilization from vegetative tissues or combined vegetative organs together to barley and wheat grain (Przulj and Momcilovic, 2001b; Abeledo et al., 2003; Sylvester-Bradley and Kindred, 2009; Dordas, 2012). The studies (Przulj and Momcilovic, 2001a,b; Dordas, 2012) have also shown a genetic variation in DM and N immobilization for barley. However, N partitioning in different plant parts has not been determined in barley, and very few studies have provided a complete N audit during grain filling in different varieties of spring malting barley; that is, how overall plant N uptake was partitioned to individual organs and subsequently remobilized to the grain to determine their roles in influencing the characteristics of malting quality.

Therefore, the objectives of this study were (i) to quantify both the genetic variation in the partitioning and remobilization of DM and N reserves from vegetative organs to the grain and post-anthesis current photosynthetic assimilation or N uptake in a selection of 23 spring malting barley varieties under recommended N fertilization conditions; (ii) to provide a benchmark audit of barley DM and N relationships in a 2-year study; and (iii) to assess the relationships among DM and N partitioning and remobilization, and grain size and protein content.

## Materials and Methods

### Field Experiments

A 2-year field experiment with 23 spring malting barley varieties in 2014 and 2015 (Table 1) was conducted at the Technical University of Munich's experimental station at Dürnast in Germany (11°41′60″E, 48°23′60″ N). Barley seeds were sown in mid-March with a seed density of 330 seeds m^−2^, and the final harvest was carried out at the end of July. A randomized complete block design with four replicates was used for the experiments. Plots consisted of 12 rows and were 10.9 m in length, i.e., 16.35 m^2^ plot^−1^. The soil is characterized as a mostly homogeneous Cambisol of silty clay loam. The residual soil mineral nitrogen (soil Nmin) to a 60-cm depth before sowing was 65 kg ha^−1^ in 2014 and 40 kg ha^−1^ in 2015. Nitrogen fertilization was applied as a dressing at 70 kg N ha^−1^ at sowing in both years based on local N recommendation. The final total N supply was 135 kg N ha^−1^ in 2014 and 110 kg N ha^−1^ in 2015.

**Table 1:** Spring malting barley varieties in 2014 and 2015 showing the name, year of release, precocity, genetic origin, breeder and country of origin (G-Germany, Aus-Australia).

			Precocity^*^				
**No**.	**Variety Name**	**Listed**	**Head emergence**	**Maturity**	**Genetic origin**	**Breeder**	**Country**
1	Aspen	1999	5	5	Vintage x Chariot	Nickerson	G
2	Barke	1996	5	5	Libelle x Alexis	Breun	G
3	Baronesse	1989	4	5	(343/6 × J-427) × (Oriol × 6153 P40)	Nordsaat	G
4	Braemar	2002	5	5	NFC 5563/NFC 94-20	NFC/Cebeco	G
5	Carina	1973	5	4	(Union x W 16 WV) x Volla	Ackermann	G
6	Grace	2008	4	5	(Xanadu x Simba) x Marnie	Ackermann	G
7	IPZ 24727	–	–	–	(Br.3546*Omega*Trumpf)*Maresi	LfL	G
8	Irina	2012	5	6	–	SKW	G
9	Mackay	2003	–	–	–	–	AUS
10	Marthe	2005	5	5	Neruda/Recept	Nordsaat	G
11	Melius	2012	5	5	Conchita * Tamtam	Syngenta	G
12	Power	1998	5	5	Saloon/(Colada/(Lux/Annabell))	–	G
13	Quench	2006	6	6	Sebastian × Drum	Syngenta/NFC	G
14	Salome	2011	5	5	(Publican × Beatrix) × Auriga	Nordsaat	G
15	Scarlett	1995	5	5	(Amazone × Br. 2730e) × Kym	Breun	G
16	Shakira	2004	–	5	–	–	G
17	Sissy	1990	–	–	(Frankengold × Mona) × Trumpf	Streng	G
18	Solist	2012	5	5	S03F049(Marnie*Simba)*S99G264	Streng	G
19	Trumpf	2003	–	–	(Diamant × 14029/64/6 (Alsa × S3170/Abyss) × 11719/59) × Union	Hadmersleben	G
20	Union	1955	–	–	(Weihenst. MR II × Donaria) × Firl. 621	Firlbeck	G
21	Ursa	2002	5	6	(Thuringia × Hanka) × Annabell	Nordsaat	G
22	Volla	1957	–	–	Wisa × Haisa I	Breun	G
23	Wiebke	1998	–	–	–	–	G

* *Numbers 4, 5, 6, and 7 indicate “early to medium,” “medium,” “medium to late,” and “late” head emergence or maturity, respectively*.

### Weather Conditions

**Figure 1:**
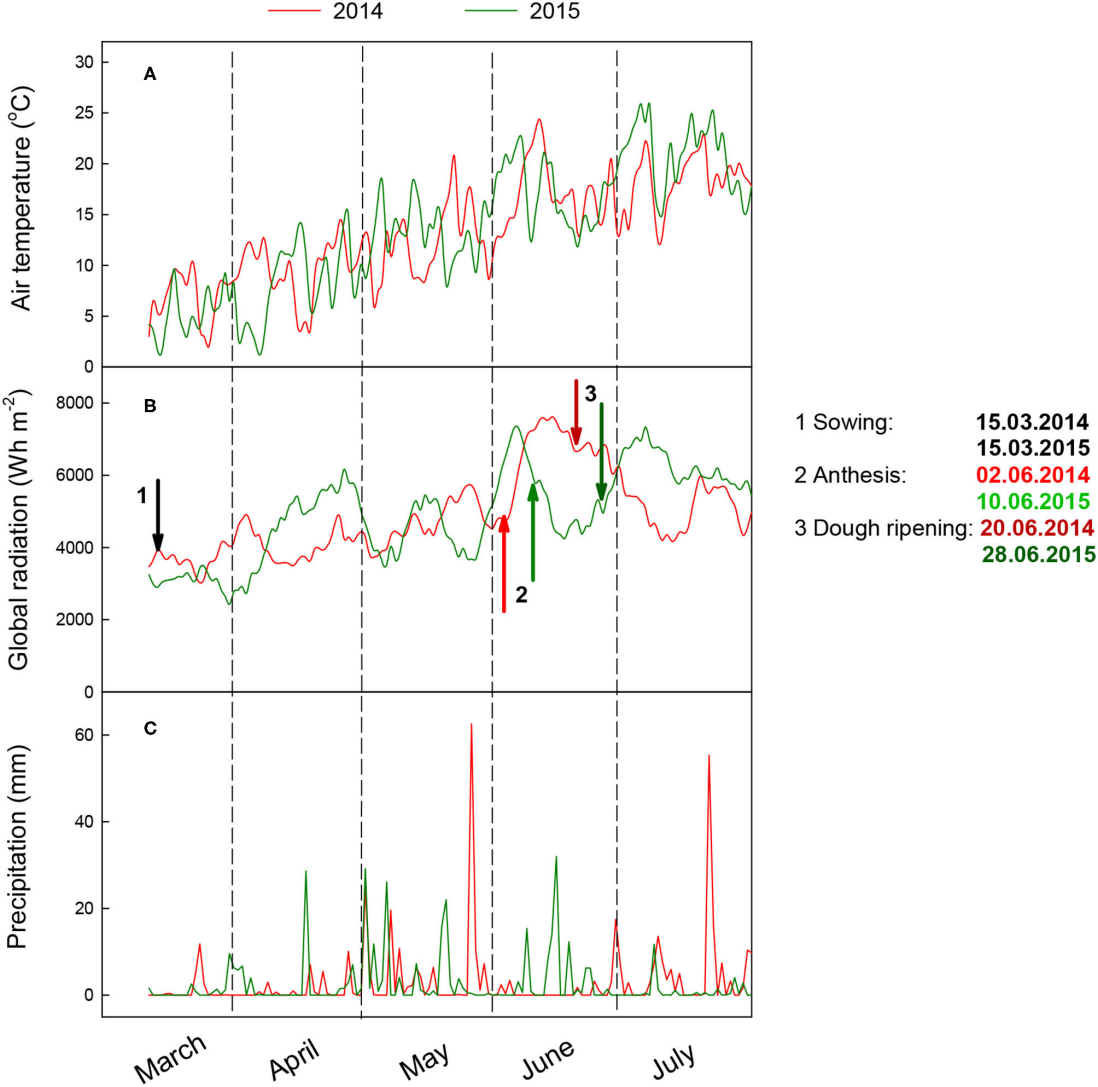
**(A)** Daily air temperature, **(B)** global radiation, and **(C)** precipitation during the growing season from March to July in 2014 and 2015 (DWD, https://www.dwd.de/). Global radiation is presented as smoothed by a 10-day moving average.

The average annual precipitation in this region is ~800 mm, and the average annual temperature is 7.8°C. The data derived from the weather station of the German Meteorological Service (DWD) next to the experimental site in 2014 and 2015 are shown in Figure 1. The year 2014 had favorable growing conditions in March with a higher temperature and more radiation than 2015 (Figure 1). However, the air temperature in both years was similar in April. In contrast to some drought periods in April in both years, there was more precipitation in May 2014 and 2015. In 2015, strong precipitation in May caused flooding in some plots. In June, there was less precipitation in 2014 than in 2015, i.e., the grain filling phase in 2014 benefited from a high radiation budget in June. Physiological maturity occurred mid-July in both years; the plants were finally harvested at the end of July.

### Measurements and Analysis

Growth stages, such as anthesis, dough ripening, and maturity, among the barley varieties were recorded (Figure 1). Among the 23 barley varieties, the anthesis stage differed by only 1–2 days in 2014 and 1 day in 2015, even though there was different precocity among the varieties reported in the literature (Table 1).

Biomass sampling was performed at anthesis (ZS65) and maturity (ZS92) (Zadoks et al., 1974) by randomly harvesting 30 representative plants across each plot by hand-cutting. At anthesis, the plants were separated into leaves, sheaths, and (stems + ears) (STER) in 2014 and 2015. The plants were separated into ears, leaves, sheaths, and stems at plant maturity, and then the barley ears were threshed into grain and chaff. The plant materials were oven-dried at 60°C for 2 days to achieve constant dry weight, and then the dry weight for individual parameters was determined. The N content of each measured DM parameter was detected by mass spectrometry using an isotope ratio mass spectrometer with an ANCA SL 20–20 preparation unit (Europe Scientific, Crewe, United Kingdom).

### Calculations of DM and N Translocation Within the Barley Plants

Remobilization or translocation of reserves of carbon assimilates and N originating from pre-anthesis assimilation, which is the net loss of DM and N of the vegetative organs from anthesis to maturity, has been calculated by different authors (e.g., Cox et al., 1985a,b; Papakosta and Gagianas, 1991). This approach assumes that decreases in aboveground vegetative biomass and N accumulation between anthesis and maturity are exclusively due to the mobilization of reserves, but respiratory losses involved with maintenance and redistribution or reserve photosynthates are not considered, and the role of the roots as a source of pre-anthesis C is ignored. The shedding of dead leaves that occurs during grain filling may overestimate the mobilization of both DM and N (Gebbing et al., 1998; Przulj and Momcilovic, 2001a,b; Dordas, 2012). DM and N translocation within the barley plants were calculated as follows:

(1) DM translocation (DMT) (kg ha^−1^) = DMi at anthesis – DMi at maturity, where i is leaf, sheath, STER at anthesis or (stem + chaff) (STCH) at maturity, and shoot total (leaf + sheath + STER) at anthesis or total aboveground vegetative organs (leaf + sheath + STCH) at maturity;
(2) total DM translocation efficiency (DMTE) (%) = (total DMT/total DM at anthesis) ^*^ 100, which is presented in Supplementary Tables 1 and 3;
(3) DM accumulation at post-anthesis (t ha^−1^) (DMPA) = total DM at maturity – total DM at anthesis;
(4) N translocation (NT) (kg ha^−1^) = Ni at anthesis – Ni at maturity, where i is leaf, sheath, STER at anthesis or STCH at maturity, and shoot total (leaf + sheath + STER) at anthesis or total aboveground vegetative organs (leaf + sheath + STCH) at maturity;
(5) N translocation efficiency (NTE) (%) = (NT/N accumulation at anthesis) ^*^ 100, which is presented in Supplementary Tables 5 and 7; and
(6) N uptake at post-anthesis (kg ha^−1^) (NPA) = total aboveground N accumulation at maturity – total N uptake at pre-anthesis.

### Statistical Analysis

Statistical analysis was performed using the SPSS software (SPSS ver. 26, IBM, Armonk, NY, United States). An ANOVA was carried out using a general linear model (GLM) in SPSS to compare the effects of variety (V) as fixed factor, year (Y) as random factor, and their interactions on partitioning and translocation traits of DM and N. The results of the ANOVA are reported to show the mean square, *P*-value, and partial Eta squared (PES) that is considered as a measure of variance components (Tables 2 and 3). Compared with the variety effect, the year effect was significant for most traits of the DM and N partitioning and translocation (Tables 2 and 3). The PES values also indicate that the year effect is the most important single factor influencing the performance of DM and N accumulation at pre-anthesis, and DM and N translocation from their reserves at pre-anthesis. The results of Tukey's tests are presented in Supplementary Tables 1–8. The degree of association between variables and malting quality parameters was estimated by Pearson correlation analysis.

**Table 2:** ANOVA (mean squares) of dry matter (DM) and nitrogen (N) accumulation in leaves, sheaths, STER or STCH at anthesis (ZS65) and maturity (ZS92), and grain and total aboveground organs of the 23 spring malting barley varieties cultivated for 2 years at the same site under recommended N fertilization conditions.

**Source of variance**	**DF**	**Anthesis (ZS65)**												**Maturity (ZS92)**														
		**Leaves**			**Sheaths**			**STER**			**Shoot total**			**Leaves**			**Sheaths**			**STCH**			**Grain**			**Above-ground organs**		
		**Mean square**	***P***	**PES**	**Mean square**	***P***	**PES**	**Mean square**	***P***	**PES**	**Mean square**	***P***	**PES**	**Mean square**	***P***	**PES**	**Mean square**	***P***	**PES**	**Mean square**	***P***	**PES**	**Mean square**	***P***	**PES**	**Mean square**	***P***	**PES**
		**Dry matter (DM)**																										
Year (Y)	1	4.8	***	0.78	3.7	***	0.90	55.7	***	0.74	52.0	***	0.62	0.14	*	0.22	0.02	ns	0.02	0.43	ns	0.06	2.21	ns	0.07	2.61	ns	0.03
Variety (V)	22	0.07	ns	0.55	0.05	*	0.73	1.15	ns	0.56	1.57	ns	0.52	0.03	ns	0.58	0.07	ns	0.59	0.98	**	0.75	1.18	ns	0.46	2.89	ns	0.44
V × Y	22	0.06	ns	0.15	0.02	ns	0.16	0.91	ns	0.16	1.43	ns	0.14	0.02	ns	0.12	0.05	ns	0.11	0.33	ns	0.12	1.39	ns	0.15	3.69	ns	0.13
		**Nitrogen (N)**																										
Year (Y)	1	1,643	***	0.61	3,157	***	0.95	29,480	***	0.91	35,104	***	0.84	2	ns	0.07	0.07	ns	0.09	14	ns	0.08	27,000	***	0.84	28,568	***	0.81
Variety (V)	22	52	ns	0.52	12	ns	0.62	212	ns	0.60	459	ns	0.60	0.7	ns	0.42	0.91	*	0.72	7.1	ns	0.50	403	ns	0.64	513	ns	0.63
V × Y	22	48	ns	0.14	7	*	0.21	141	ns	0.18	307	ns	0.14	1.0	ns	0.17	0.36	ns	0.11	7.1	ns	0.16	227	ns	0.11	305	ns	0.11

Statistically significant differences are indicated: *P < 0.05; **P < 0.01, ***P < 0.001 and ns, not significant. The partial Eta squared (PES) is considered as a measure of variance components.

**Table 3:** Analysis of variance (mean squares) of translocation of leaves, sheaths, STER from pre-anthesis and DM accumulation or N uptake of grains from post-anthesis of the 23 spring malting barley varieties cultivated for 2 years at the same site under recommended N fertilization conditions.

		**Translocation**												**DM or N accumulation at post-anthesis**		
**Source of variance**	**DF**	**Leaves**			**Sheaths**			**STER**			**Shoot total**					
		**Mean square**	***P***	**PES**	**Mean square**	***P***	**PES**	**Mean square**	***P***	**PES**	**Mean square**	***P***	**PES**	**Mean square**	***P***	**PES**
		**Dry matter (DM)**														
Year (Y)	1	3.29	***	0.76	4.34	***	0.80	46.4	***	0.81	50.2	***	0.80	31.4	***	0.52
Variety (V)	22	0.03	ns	0.37	0.07	ns	0.57	0.76	ns	0.60	0.74	ns	0.57	1.35	ns	0.51
V x Y	22	0.05	*	0.22	0.05	ns	0.15	0.51	ns	0.20	0.56	ns	0.18	1.30	ns	0.15
		**Nitrogen (N)**														
Year (Y)	1	1,746	***	0.67	3,187	***	0.96	28,202	***	0.92	33,339	***	0.87	336	ns	0.11
Variety (V)	22	45	ns	0.53	9.0	ns	0.57	176	ns	0.60	352	ns	0.61	207	ns	0.62
V x Y	22	40	ns	0.14	6.7	*	0.23	119	ns	0.19	228	ns	0.14	129	ns	0.19

Statistically significant differences are indicated: *P < 0.05; ***P < 0.001 and ns, not significant. The partial eta squared (PES) is considered as a measure of variance components.

## Results

### Dry Matter Accumulation, Partitioning, and Translocation

Analysis of variance revealed that, except for the DM of the sheath at ZS65 and STCH at ZS92, there was no significant difference in the measured DM parameters at anthesis (ZS65) and maturity (ZS92) among the varieties (Table 2). Although differences between the 2 years were generally significant for the DM of leaves, sheaths, and STER at anthesis, there was no significant difference in the measured DM parameters at maturity except for leaves. The year × variety interaction was not significant for all the measured DM parameters at either anthesis or maturity. The PES values shown in Table 2 indicate that the year effect is the most important single factor influencing the performance of DM at pre-anthesis.

The analysis of variance for the amount of DM translocation from vegetative organs during grain filling (DM in ZS 65 minus DM in ZS 92) (Table 3) showed that the variety effect was not significant for DM remobilization traits. The year effect was significant for DM translocation from all vegetative organs, while the V × Y interactive effect was only significant for DM translocation of the DM reserves of leaves at anthesis. The DM accumulation from post-anthesis was significant between the 2 years, whereas there were no significant effects of genotypes and interactive effects of genotypes × years for post-anthesis DM contributing to grain yield (Table 3). The PES values shown in Table 3 indicate that the year effect is the most important single factor influencing the performance of DM translocation from their reserves at pre-anthesis.

**Figure 2:**
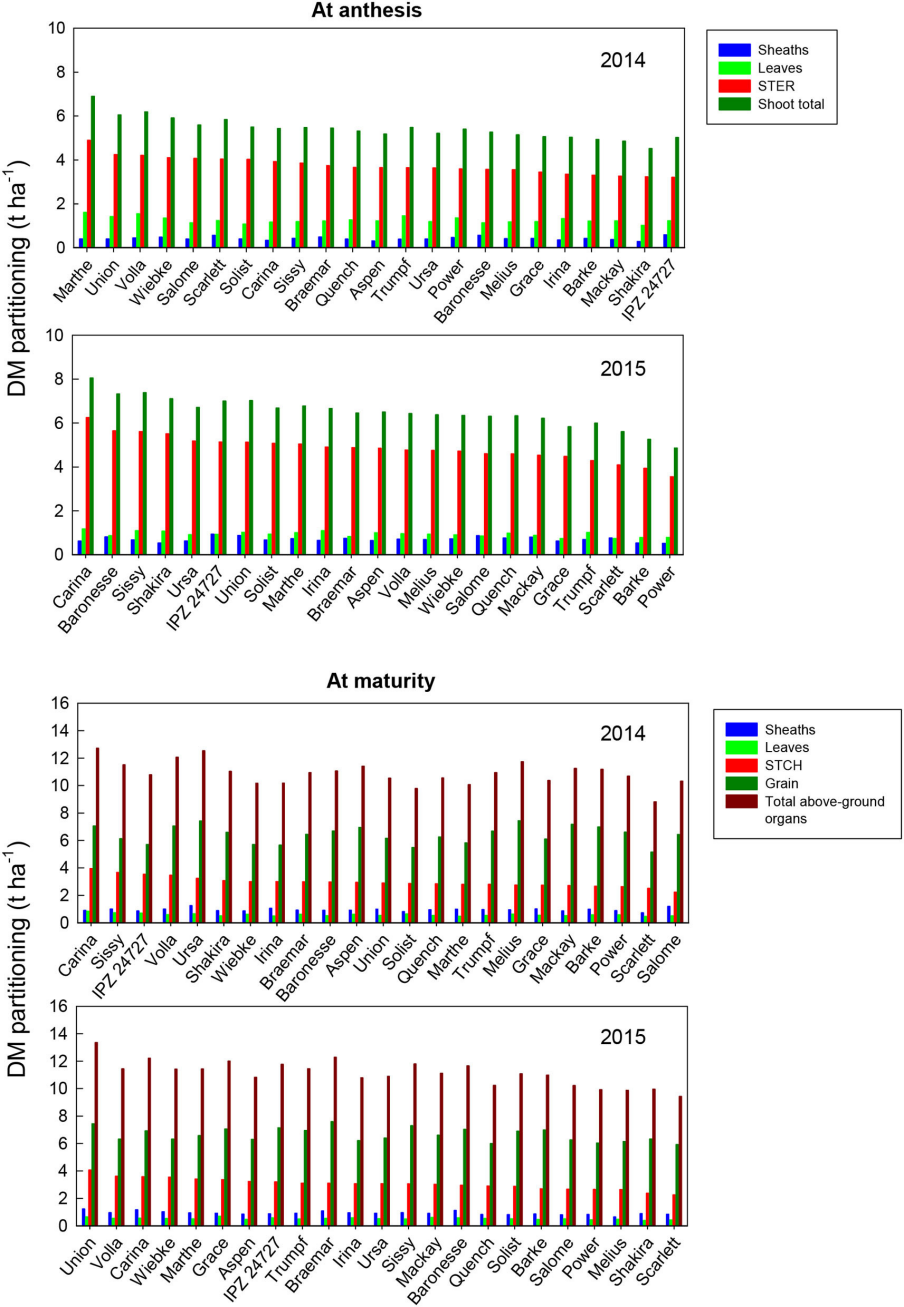
Dry matter partitioning in different organs: dry matter (DM) accumulation (t ha^−1^) of different plant organs of the 23 spring malting barley varieties at anthesis and maturity under recommended N fertilization conditions in 2014 and 2015, ranked on STER DM at anthesis or STCH DM at maturity. Mean comparisons among the varieties from Tukey's honestly significant difference (HSD) test are shown in Supplementary Tables 1 and 3.

The partitioning of DM in individual organs (leaf blades, sheaths, STER) of the fertile shoots of the 23 barley varieties at anthesis and maturity is shown in Figure 2. The total shoot DM at anthesis, averaged over all varieties, was 5.4 t ha^−1^ in 2014 and 6.5 t ha^−1^ in 2015. This was distributed in the order: STER (69%) > leaves (23%) > sheaths (8%) in 2014 and STER (75%) > leaves (14%) > sheaths (11%) in 2015. Total shoot DM for different varieties ranged from 4.5 t ha^−1^ (Shakira) to 6.9 t ha^−1^ (Marthe) in 2014, and from 4.9 (Power) to 8 t ha^−1^ (Carina) in 2015. Leaf DM for the different varieties ranged from 1 (Shakira) to 1.6 t ha^−1^ (Marthe) in 2014, and from 0.7 (Scarlett) to 1.2 t ha^−1^ (IPZ 24727) in 2015. Sheath DM for the different varieties ranged from 0.3 (Shakira) to 0.59 t ha^−1^ (IPZ 24727) in 2014, and from 0.5 (Power) to 0.9 t ha^−1^ (IPZ 24727) in 2015. STER DM for different varieties ranged from 3.2 (IPZ 24727) to 4.9 t ha^−1^ (Marthe) in 2014, and from 3.5 (Power) to 6.2 t ha^−1^ (Carina) in 2015. In addition to sheath DM accumulation, there is no significant difference in leaf and STER DM accumulation among the varieties in 2014 (Supplementary Table 1), and no significant difference among the varieties is found in leaf DM accumulation in 2015 (Supplementary Table 3).

The total shoot DM at maturity, averaged over all varieties, was 10.9 t ha^−1^ in 2014 and 11.1 t ha^−1^ in 2015 (Figure 2). This was distributed in the order: grain (59%) > STCH (27%) > sheaths (9%) > leaves (5%) in 2014, and grain (60%) > STCH (27%) > sheaths (8%) > leaves (5%) in 2015. Total shoot DM for different varieties ranged from 8.8 (Shakira) to 12.7 t ha^−1^ (Union) in 2014, and from 9.4 (Salome) to 13.3 t ha^−1^ (Carina) in 2015. The leaf DM of the different varieties ranged from 0.45 (Shakira) to 0.84 t ha^−1^ (Union) in 2014, and from 0.38 (Scarlett) to 0.68 t ha^−1^ (Shakira) in 2015. The sheath DM of the different varieties ranged from 0.7 (Shakira) to 1.2 t ha^−1^ (Marthe) in 2014, and from 0.6 (Power) to 1.2 t ha^−1^ (Carina) in 2015. STCH DM for different varieties ranged from 2.2 (Scarlett) to 3.9 t ha^−1^ (Union) in 2014, and from 2.2 (Salome) to 4.1 t ha^−1^ (Carina) in 2015. Grain DM for different varieties ranged from 5.1 (Shakira) to 7.4 t ha^−1^ (Marthe) in 2014, and from 5.9 (Salome) to 7.6 t ha^−1^ (Baronesse) in 2015. There was no significant difference in DM accumulation of all the organs measured among the varieties either in 2014 or 2015 (Supplementary Tables 1 and 3).

**Figure 3:**
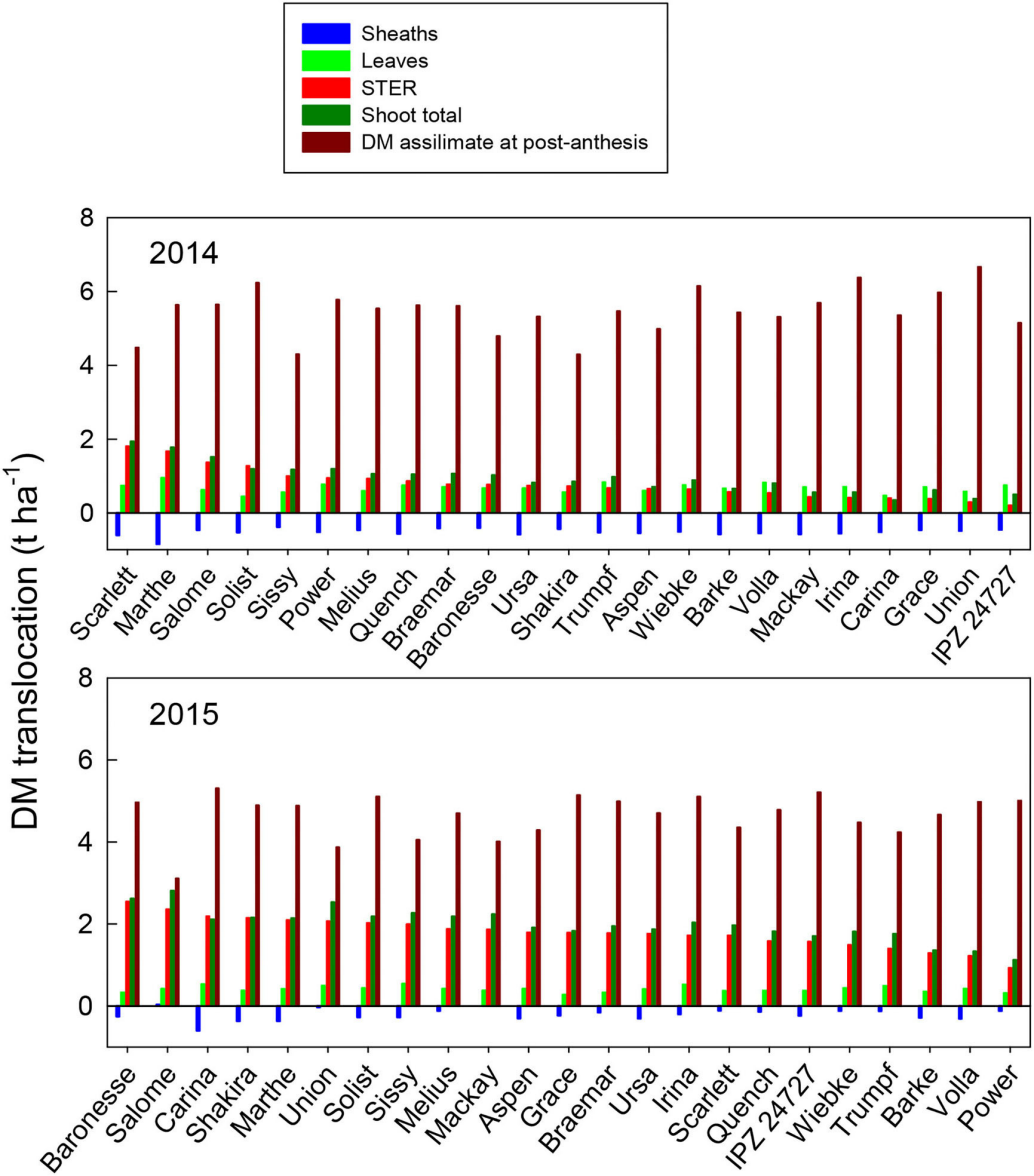
Dry matter translocation from different organs: DM translocation (t ha^−1^) of different plant organs of the 23 spring malting barley varieties to grain from pre-anthesis and maturity under recommended N fertilization conditions in 2014 and 2015, ranked on DM translocation of STER. Mean comparisons among the varieties from Tukey's HSD test are shown in Supplementary Tables 2 and 4.

The amount of DM translocation from vegetative organs during grain filling (ZS 65 minus ZS 92) from the different varieties ranges from 0.35 (Carina) to 1.9 t ha^−1^ (Scarlett) in 2014, and from 1.1 (Power) to 2.8 t ha^−1^ (Salome) in 2015 (Figure 3). This calculation assumed that all post-anthesis photosynthetic assimilates went directly to the grains. Total translocation from the DM reserves in vegetative organs at pre-anthesis accounts for 6–29% of the contribution to grain DM among the varieties in 2014 and 19–47% in 2015 (Figure 3). STER at ZS 65 and STCH at ZS 92 were the dominant contributors to this transfer in 2015. In contrast, the reserve from leaf DM contributed similarly to that from STER at pre-anthesis for most of the varieties in 2014. The difference in the total shoot translocation among the barley varieties in 2014 was significant, whereas no significant difference was found in 2015 (Supplementary Tables 2 and 4). The amount of post-anthesis DM accumulation in grains from different varieties ranged from 4.3 (Shakira) to 6.7 t ha^−1^ (Union) in 2014, and from 3.1 (Salome) to 5.3 t ha^−1^ (Carina) in 2015 (Figure 3). The post-anthesis assimilates accounted for 71–94% of the total grain yield among the barley varieties in 2014 and 53–81% in 2015. However, there was no significant difference in post-anthesis DM assimilation in either year (Supplementary Tables 2 and 4).

### Nitrogen (N) Accumulation, Partitioning, and Translocation

Analysis of variance reveals that at anthesis, there is no significant difference in N partitioning among the genotypes except for sheath N at maturity (Table 2). The year effect was significant for N reserves in all measured vegetative organs at anthesis, whereas the significant year effect was found only for grain N and total N at maturity. The variety × year interaction was only found for sheath N at anthesis. The PES values shown in Table 2 indicate that the year effect is the most important single factor influencing the performance of N accumulation at pre-anthesis.

**Figure 4:**
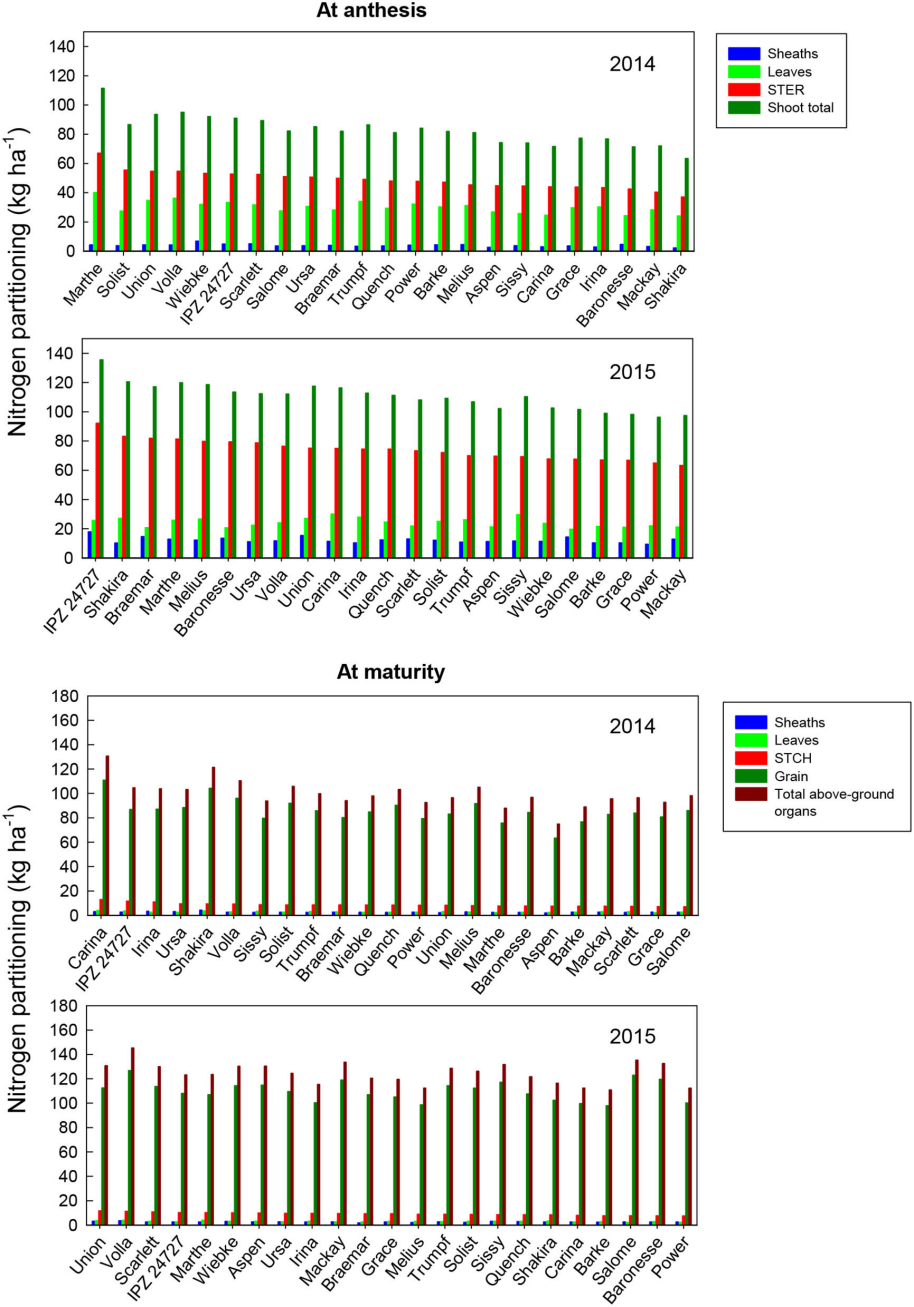
Nitrogen partitioning in different organs: N accumulation (kg ha^−1^) of different plant organs of the 23 spring malting barley varieties at anthesis and maturity under recommended N fertilization conditions in 2014 and 2015, ranked on STER N at anthesis or STCH N at maturity. Mean comparisons among the varieties from Tukey's HSD test are shown in Supplementary Tables 5 and 7.

The analysis of variance for the amount of N translocation from vegetative organs during grain filling (N reserves at ZS 65 minus at ZS 92) (Table 3) showed that there was no significant difference in the N remobilization from the sheaths (stems + ears) and shoot total at anthesis among the genotypes. The year effect showed a significant difference in N from all individual organs. The variety × year interactive effects were only significant for sheath N translocation. The N uptake for grains post-anthesis is not significant between the 2 years, among the varieties or for the variety × year interaction (Table 3). The PES values shown in Table 3 indicate that the year effect is the most important single factor influencing the performance of N translocation from their reserves at pre-anthesis. The partitioning of nitrogen (N) in individual organs (leaf blades, sheaths, STER) of the fertile shoots of the 23 barley varieties at anthesis and maturity is shown in Figure 4. The total shoot N at anthesis, averaged over all the varieties, was 83 kg ha^−1^ in 2014 and 111 kg ha^−1^ in 2015. This was distributed in the order: STER (59%) > leaves (36%) > sheaths (5%) in 2014, and STER (67%) > leaves (22%) > sheaths (11%) in 2015. The total shoot N for the different varieties ranged from 63 (Sharika) to 111 kg ha^−1^ (Marthe) in 2014, and from 96 (Power) to 136 kg ha^−1^ (IPZ 24727) in 2015. Leaf N for the different varieties ranged from 24 (Sharika) to 40 kg ha^−1^ (Marthe) in 2014, and from 20 (Salome) to 30 kg ha^−1^ (Carina) in 2015. Sheath N for the different varieties ranged from 2 (Sharika) to 7 kg ha^−1^ (Wiebke) in 2014, and from 9 (Power) to 18 kg ha^−1^ (IPZ 24727) in 2015. STER N for the different varieties ranged from 37 (Sharika) to 67 kg ha^−1^ (Marthe) in 2014, and from 63 (Mackay) to 92 kg ha^−1^ (IPZ 24727) in 2015. Besides leaf DM accumulation, there was a significant difference in sheaths and STER N accumulation between the varieties in 2014 (Supplementary Table 5), while no significant difference between the varieties was found in leaf and STER N accumulation in 2015 (Supplementary Table 7).

The total shoot N at maturity, averaged over all the varieties, was 100 kg ha^−1^ in 2014 and 125 kg ha^−1^ in 2015 (Figure 4). This was distributed in the order: grain (86%) > STCH (8.5%) > leaves (2.7%) > sheaths (2.6%) in 2014, and grain (88%) > STCH (7.4%) > leaves (2.3%) > sheaths (2%) in 2015. The total shoot N for the different varieties ranged from 75 (Sharika) to 131 kg ha^−1^ (Union) in 2014, and from 111 (Mackay) to 145 kg ha^−1^ (IPZ 42727) in 2015. Leaf N for the different varieties ranged from 2 (Baronesse) to 4 kg ha^−1^ (Union) in 2014, and from 2 (Scarlett) to 4 kg ha^−1^ (IPZ 42727) in 2015. Sheath N for the different varieties ranged from 2 (Shakira) to 4 kg ha^−1^ (Marthe) in 2014, and from 1.8 (Wiebke) to 3.4 kg ha^−1^ (IPZ 42727) in 2015. STCH N for the different varieties ranged from 7 (Power) to 13 kg ha^−1^ (Union) in 2014, and from 7 (Salome) to 12 kg ha^−1^ (Carina) in 2015. Grain N for the different varieties ranged from 63 (Sharika) to 111 kg ha^−1^ (Union) in 2014, and from 98 (Power) to 127 kg ha^−1^ (IPZ 42727) in 2015. In addition to the sheath N accumulation in 2014, there was no significant difference in measured plant organs at ZS 92 among the varieties in 2014 and 2015 (Supplementary Tables 5 and 7).

**Figure 5:**
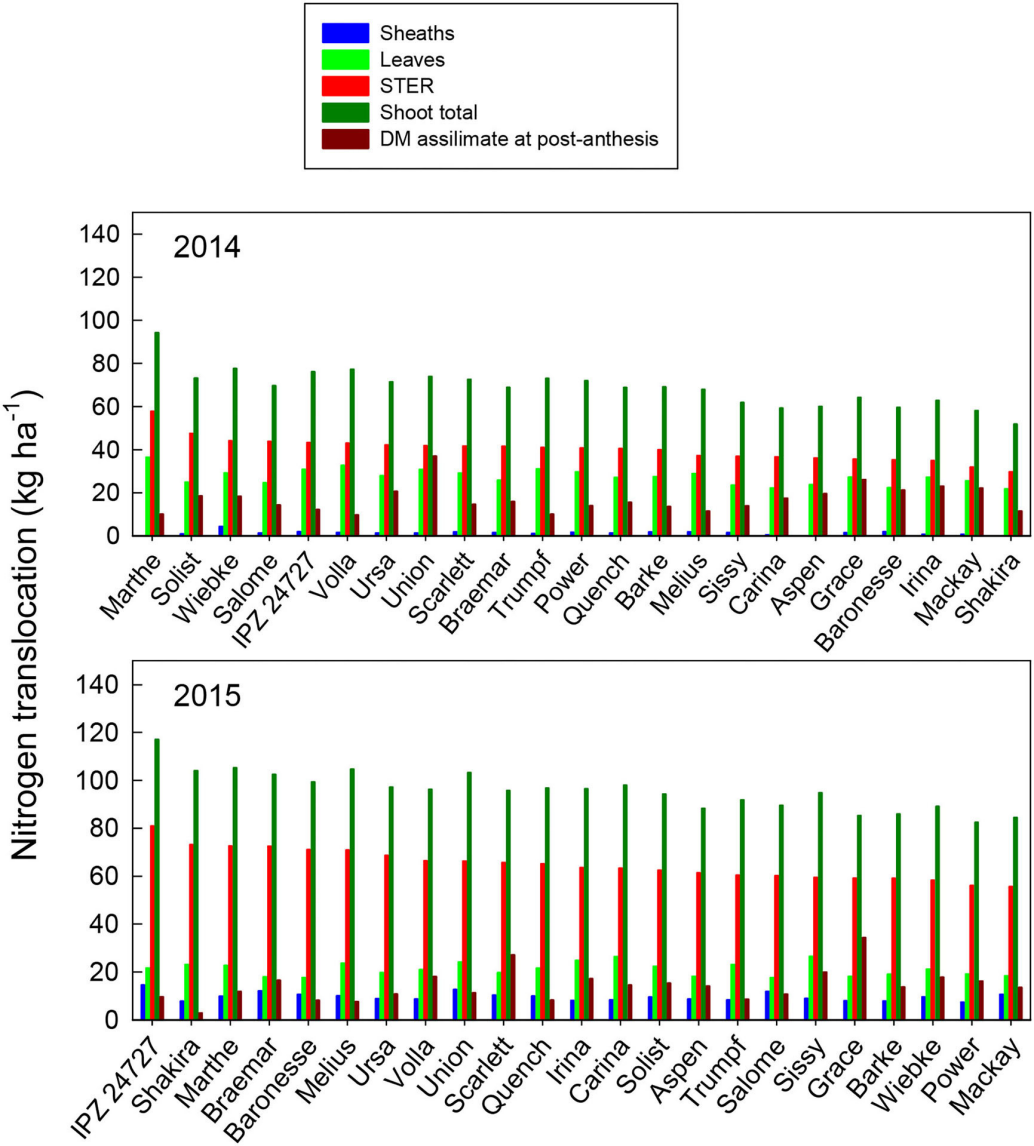
Nitrogen translocation from different organs: N translocation (kg ha^−1^) of different plant organs of the 23 spring malting barley varieties to grain from pre-anthesis and maturity under recommended N fertilization conditions in 2014 and 2015, ranked on N translocation of STER. Mean comparisons among varieties from Tukey's HSD test are shown in Supplementary Tables 6 and 8.

The amount of N translocation from vegetative organs during grain filling (N accumulation at ZS 65 minus at ZS 92) of different varieties ranged from 52 (Shakira) to 94 kg ha^−1^ (Marthe) in 2014, and from 82 (Power) to 117 kg ha^−1^ (IPZ 42727) in 2015 (Figure 5). The contribution of N translocation from pre-anthesis to grain N varied, ranging from 67 (Union) to 91% (Marthe) in 2014, and 71% (Grace) to 97% (Sharika) in 2015. However, a significant difference among the varieties was only found in 2014 (Supplementary Tables 6 and 8). Similar to DM translocation, the N reserves from STER were the dominant contributor to this transfer. The amount of post-anthesis N uptake of grains among the 23 varieties ranged from 9.7 (Volla) to 37 kg N ha^−1^ (Union) in 2014, and from 2.9 (Sharika) to 34 kg N ha^−1^ (Grace) in 2015 (Figure 5). The post-anthesis N uptake accounted for 9–33% of the total grain N among the barley varieties in 2014, and for 3–29% in 2015. A significant difference in the post-anthesis N uptake of grains was found in both years (Supplementary Tables 6 and 8).

## Discussion

In this study, the most important single factor influencing the performance of dry matter and nitrogen accumulation at pre-anthesis and dry matter and nitrogen partitioning and translocation from their reserves at pre-anthesis was the year (Tables 2 and 3). The year effect could be due to the variation in weather conditions, such as precipitation and temperature, between the 2 years (Figure 1). Figures 2–5 show that the ranking of the 23 barley varieties based on DM and N parameters is inconsistent with the DM and N parameters for a given year and varied with the year for the same barley variety. Abeledo et al. (2008) reported that modern malting barley cultivars tended to have a higher N content in ears at pre-anthesis than old cultivars. However, this tendency was not observed in this study. For example, Figure 4 shows that cultivars registered after the 2000s had both higher and lower N contents (kg ha^−1^) in plant organs at pre-anthesis in 2014 and 2015 (Table 1). Furthermore, although Figures 2–5 demonstrate genetic variation for most parameters, most parameter differences were not statistically significant (Supplementary Tables 1–8). A similar tendency was also found for the grain quality properties of the grain protein content and the grain retention fraction of grain size > 2.5 mm (Hu et al., 2021). Most importantly, because the N partitioning in the different plant parts in barley has not been reported in the literature, the results in this study can provide a benchmark audit of the DM and N accumulation in different organs at pre- and post-anthesis in a 2-year study, which allows us to first discuss the physiological basis of genetic variation in DM and N partitioning, and DM and N remobilization, and shows source-sink relationships and their responses to the year effect. Second, a better understanding of the roles of DM and N partitioning and remobilization in determining the quality properties of spring malting barley may help to identify their roles in determining malting quality, which will allow us to develop strategies for trait selection and agronomic management to obtain suitable grain size and protein content that can ensure meeting the requirements from the malting and brewing industry.

### Partitioning and Translocation of Dry Matter and Their Roles in Determining Malting Quality

Earlier studies on barley have reported that DM remobilization from reserves in vegetative organs at pre-anthesis to grains reached ~ 4–24 (Przulj and Momcilovic, 2001a,b) and 12–28% (Dordas, 2012). In this study, the DM stored in vegetative tissues at anthesis contributed to grain yield from 6 in the variety Union to 29% in the variety Scarlett in 2014, and from 19 in the variety Power to 47% in the variety Salome in 2015 (Figure 2). The values in 2014 were similar to those reported in the literature, whereas the values in 2015 were much higher than those in this study from 2014 and the other studies cited above, indicating that fewer photosynthetic assimilates were produced during grain filling in 2015, probably because of faster senescence. Figure 3 demonstrates a higher DM assimilate at post-anthesis in 2014 than that in 2015. More interestingly, the varieties with a longer period from heading and maturity (Table 1) such as Baronesse, Grace, Irina, and Ursa showed a higher rank among the 23 varieties based on the DM translocation in 2015 than in 2014. With regard to DM translocation, DM reserves in leaves and stems at pre-anthesis represent the major contribution to barley grain yield (Przulj and Momcilovic, 2001a). In wheat, the cultivars with more assimilates stored in the stem and greater assimilative capacity of ears, especially a greater contribution of ear assimilates, are expected to increase the grain yield (Sun et al., 2021). Elazab et al. (2021) suggested that to develop winter wheat (*Triticumaestivum* L.) breeding strategies, the increase in ear size plays an important role in photosynthetic assimilates for grain filling. In this study, the average DM translocation from leaves among all the barley varieties at pre-anthesis was similar to that from stems and ears in 2014, whereas this was 5-fold higher than that from leaves in 2015. Merah et al. (2018) reported that the contribution of ear photosynthesis and re-mobilization from the stem in durum wheat increased with post-anthesis water stress. Furthermore, this study found that the DM translocation from sheaths at pre-anthesis was negative in both years (Figure 3). In contrast, less negative values were found in 2015 than in 2014. This may be due to the barley's earlier flowering in 2014 than in 2015 (Figure 1).

Barley growth and development before anthesis determine the grain sink capacity, which is a function of the number of grains per unit land area and their potential size (individual storage capacity). Post-anthesis photosynthetic activity and the remobilization of soluble carbohydrate reserves stored from pre-anthesis supply carbon assimilates during grain filling (Kennedy et al., 2017; Bingham et al., 2019). The balance between the source and sink capacity during grain filling varies depending on environmental conditions and season (Grashoff and d'Antuono, 1997; Borras et al., 2004). This study showed that the post-anthesis assimilates accounted for 71–94% of the total grain yield among the barley varieties in 2014 and 53–81% in 2015, which is in agreement with more sink- than source-limiting for grain filling (Borras et al., 2004). Hu et al. (2021) reported that the grain number per ha in 2015 was higher than that in 2014, indicating a higher sink potential in 2015 for carbon assimilates. However, the grain retention fraction >2.5 mm from the 23 barley varieties was <90% (Hu et al., 2021), which could not meet the malting barley quality requirement of the brewing industry in the West European market. In contrast, the grain retention fraction >2.5 mm was > 90% for almost all the barley varieties tested in 2014. This may suggest that the photosynthetic activity in 2015 was a limiting factor during grain filling, i.e., source was limited. The results of correlations among the grain retention fraction and DM accumulation at pre- and post-anthesis and translocation across the 2 years shown in Tables 4, 5 demonstrate that the grain retention fraction was significantly associated with leaf DM accumulation at pre-anthesis and photosynthetic assimilates for grains during grain filling, while there was a negative correlation among the grain retention fraction and DM accumulation in sheaths and STER at pre-anthesis and translocation from sheaths and STER. This further suggests that photosynthetic activity for high photosynthetic assimilates at post-anthesis may determine barley grain size, suggesting that this is an important factor in improving malting quality. The awns of the ear have been reported as important sources of assimilates in wheat. Studies by Maydup et al. (2014) and Merah and Monneveux (2015) have shown that there is a positive relationship between awn size and contribution of the ear to grain filling under stress conditions, and that chaff and awns were even better correlated with grain than stem and leaf. According to the studies on wheat by Merah and Monneveux (2015) and Elazab et al. (2021), carbon isotope discrimination is a rapid and non-destructive technique for the estimation of variations in the contribution of different organs to grain filling. To gain a better understanding of increased ear photosynthesis, manipulation techniques such as carbon isotope discrimination are needed.

**Table 4:** Pearson's correlation coefficient of grain protein content and grain size with DM and N accumulation in leaves, sheaths, STER or STCH, and total aboveground organs at anthesis and maturity and grain yield among the 23 spring malting barley varieties under the recommended N fertilization conditions across 2 years.

	**Grain protein content**				**Grain size**			
	**Anthesis**		**Maturity**		**Anthesis**		**Maturity**	
**Dry matter of**								
Leaves	−0.62	**	−0.17	ns	0.64	**	0.20	ns
Sheaths	0.80	**	0.07	ns	−0.78	**	0.04	ns
STER or STCH	0.70	**	0.34	*	−0.73	**	−0.24	ns
Grains	–		0.04	ns	–		−0.19	ns
Shoot total or above-ground organs	0.66	**	0.17	ns	−0.68	**	−0.20	ns
**N accumulation in**								
Leaves	−0.49	**	0.31	*	0.54	**	−0.26	ns
Sheaths	0.87	**	0.12	ns	−0.86	**	0.05	ns
STER or STCH	0.83	**	0.38	**	−0.83	**	−0.29	*
Grains	–		0.83	**	–		−0.74	**
Shoot total or above-ground organs	0.80	**	0.83	**	−0.78	**	−0.73	**

Statistically significant differences are indicated: *P < 0.05; **P < 0.01 and ns, not significant.

**Table 5:** Pearson's correlation coefficient of grain protein content and grain size with the translocation of DM and N accumulation in leaves, sheaths and STER from pre-anthesis, and assimilation and N accumulation from post-anthesis among the 23 spring malting barley varieties under recommended N fertilization conditions across 2 years.

	**Grain protein content**				**Grain size**			
	**Dry matter (DM)**		**Nitrogen (N)**		**Dry matter (DM)**		**Nitrogen (N)**	
Leaves	−0.69	**	−0.54	**	0.70	**	0.58	**
Sheaths	0.66	**	0.86	**	−0.71	**	−0.87	**
STER	0.60	**	0.82	**	−0.70	**	−0.83	**
Shoot total	0.61	**	0.80	**	−0.71	**	−0.80	**
Post-anthesis DM accumulation or N uptake	−0.54	**	−0.04	ns	0.51	**	0.24	ns

Statistically significant differences are indicated: **P < 0.01 and ns, not significant.

### Nitrogen Partitioning and Translocation, and Their Roles in Determining Malting Quality

Nitrogen uptake and translocation play a major role in determining grain protein content. Several studies have shown that the amount of N uptake in the aboveground parts of barley and wheat crops from pre-anthesis accounts for up to 90% of the total grain N at maturity, depending on the variety and environment (e.g., Clarke et al., 1990; Heitholt et al., 1990; Przulj and Momcilovic, 2001a,b; Dordas, 2012). In this study, the N reserved in vegetative tissues at anthesis contributed to barley grain N from 67 in the variety Union to 91% in the variety Marthe in 2014, and from 71 in the variety Grace to 97% in the variety Shakira in 2015. The results of correlations among the grain retention fraction and DM accumulation at pre- and post-anthesis and translocation across the 2 years in Tables 4, 5 show that the grain protein content was significantly associated with sheath and STER N accumulation at pre-anthesis and translocation from sheaths and STER, suggesting that N uptake before anthesis may play an important role in determining the barley grain protein content.

The contribution of translocated N from the N reverses in vegetative tissue at pre-anthesis to grain N can indicate growing conditions and N availability in the soil during vegetation, i.e., higher N translocation indicates good growing conditions and the availability of N from the sources before anthesis. Hu et al. (2021) reported that grain protein content varied significantly within a year for the same barley varieties, and that average protein content was lower in 2014 than in 2015. For example, in 2015, among the 23 barley varieties, 22 obtained a protein content between 9.5 and 11.5% despite a lower N supply rate in; while in 2014, the protein content among the 23 varieties ranged from 8.3 to 9.8%, and only two varieties reached a value higher than 9.5%, which is the critical level for malting quality requirement. The lower protein content of barley grain in 2014 may reflect a lower N supply rate that may be derived from less N mineralization in 2014 during the vegetative growth because the N fertilization rate was the same for both years. Studies have shown that N level increases N concentration in plant tissue and affects dry matter and N accumulation, partitioning, and translocation (Dordas, 2012). Thus, barley needs to receive an adequate amount of N, as this affects dry matter and N translocation. Furthermore, N uptake is influenced by the available water (Clarke et al., 1990), degree of association between the roots and soil, and supply of nitrate (Cox et al., 1985a,b; Papakosta and Gagianas, 1991). This may indicate unfavorable growth conditions before anthesis in 2014 compared with 2015. Furthermore, because of the negative relationships between grain yield (and/or grain size) and protein content, protein grain content concentration in the grain depends not only on the N amount in the grain but also on the level of photosynthetic activity at post-anthesis during grain filling (Cox et al., 1986). Higher photosynthetic assimilates at post-anthesis that caused a dilution effect on grain protein content in 2014 may also be a reason for lower grain protein content (Figure 3).

Figures 4, 5 show that at anthesis, most plant N is present in stems and ears, and then in leaf blades and leaf sheaths. Stems have long been identified as a major N pool for mobilization (e.g., Gregory et al., 2013), although in most studies, “stems” also include sheaths. Thus, the results indicate that an important target to increase N reserves in stems at pre-anthesis as a selection trait for malting barley varieties can lead to enhanced N translocation from non-photosynthetic organs. Consequently, this also results in an increase in N use efficiency. In contrast, maintenance of the N level in green leaves, i.e., a delay in leaf senescence during grain filling, may contribute to more photosynthetic assimilates at post-anthesis.

In this study, the findings of the most important effect of the year as an environmental factor on the performance of barley DM and N accumulation and translocation (Tables 2 and 3) is in agreement with studies by Therrien et al. (1994), Eagles et al. (1995) and Laidig et al. (2017). Therrien et al. (1994) showed that, compared with genetic effects, environmental effects were a dominant factor in determining malting quality, and suggested the use of management practices to optimize malting barley quality, i.e., nitrogen had the greatest effect on malting quality traits. Laidig et al. (2017) reported that barley genetic variation accounted for only 3% of the total variation; thus, the environment and crop management, particularly nitrogen supply, are mainly responsible for the variation of the protein level in barley grain quality. In a simplified way, rapid on-farm tests of mineral nitrogen content at the beginning of the season will allow for the determination of residual mineral nitrogen content (Schmidhalter, 2005). To further optimize N management, sensing technology is currently available for in-season N fertilization of field crops, since this not only allows the detection of the actual growth and N status but can also be used to estimate soil nitrogen mineralization (Schmidhalter et al., 2006). Spectral sensing techniques can be used for a more targeted N application in spring barley (Barmeier and Schmidhalter, 2017; Barmeier et al., 2017).

## Conclusions

This study showed that the year effect was the most important single factor influencing the performance of DM and N accumulation at pre-anthesis and DM and N translocation from their reserves at pre-anthesis (Tables 2 and 3), which may be due to the variation in weather conditions such as precipitation and temperature between the 2 years. Post-anthesis assimilates contributed to grain yield, accounting for 71–94% among the barley varieties in 2014 and 53–81% in 2015. In contrast, the contribution of N reserved in vegetative tissues from pre-anthesis to grain N accounted for 67–91% in 2014 and 71–97% in 2015. The positive correlations among post-anthesis assimilation, grain size and N reserves at pre-anthesis, and N remobilization and grain protein content of malting barley suggest that DM and N partitioning and remobilization played an important role in determining the quality properties of spring malting barley. To achieve a high quality of malting barley grains in both grain size and protein content simultaneously, parallel strategies of optimized trait selection of spring malting barley and improved agronomic N management for higher photosynthetic activity at post-anthesis and an increase in N reserves at pre-anthesis have to be developed. Improved management strategies should include the detection of residual soil mineral nitrogen at the beginning of the season as well as the detection of soil N mineralization to optimize the production of spring malting barley.

## Data Availability Statement

The original contributions presented in the study are included in the article/Supplementary Material, further inquiries can be directed to the corresponding author.

## Author Contributions

US and GB conceived and designed the experiments. GB performed the experiments. GB and YH analyzed the data. YH and US wrote the article. All authors contributed to the article and approved the submitted version.

## Conflict of Interest

The authors declare that the research was conducted in the absence of any commercial or financial relationships that could be construed as a potential conflict of interest.

## Publisher's Note

All claims expressed in this article are solely those of the authors and do not necessarily represent those of their affiliated organizations, or those of the publisher, the editors and the reviewers. Any product that may be evaluated in this article, or claim that may be made by its manufacturer, is not guaranteed or endorsed by the publisher.
