# Supplementary material for: Partitioning and Translocation of Dry Matter and Nitrogen During Grain Filling in Spring Barley Varieties and Their Roles in Determining Malting Quality
Source: Front Plant Sci. 2021 Aug 23;12:722871. doi: 10.3389/fpls.2021.722871 (PMC8419453; doi:10.3389/fpls.2021.722871)
Supplement: Supplementary file 1 [file Data_Sheet_1.pdf]

Supplementary Table 1 Dry matter (DM) partitioning of 23 spring malting barley varieties under the optimal N fertilization in 2014. Mean (± SE) and Tukey-test for DM accumulation of leaves, sheaths, (stems+ears) or (stems+chaff) at anthesis and at maturity, and grain and total aboveground DM of spring malting barley varieties. Mean comparisons among varieties from Tukey´s HSD test indicates a significant difference at $p < 0.05$. The same letters mean groups in a column that were not significantly different from one another.

Variety Name	Dry matter partitioning (t ha^{-1})																			
	Anthesis								Maturity											
	Leaves		Sheaths		Stems+Ears		Shoot total		Leaves		Sheaths		Stems+Chaff		Straw		Grain		Aboveground organs	
	mean±SE		mean±SE		mean±SE		mean±SE		mean±SE		mean±SE		mean±SE		mean±SE		mean±SE		mean±SE	
Aspen	1.22 ± 0.06	a	0.31 ± 0.03	ab	3.65 ± 0.34	a	5.18 ± 0.37	a	0.62 ± 0.05	a	0.85 ± 0.08	a	2.99 ± 0.20	a	4.47 ± 0.31	a	5.69 ± 0.41	a	10.16 ± 0.69	a
Barke	1.21 ± 0.14	a	0.42 ± 0.02	bc	3.30 ± 0.49	a	4.93 ± 0.65	a	0.55 ± 0.07	a	1.00 ± 0.15	a	2.73 ± 0.33	a	4.27 ± 0.54	a	6.09 ± 0.71	a	10.36 ± 1.24	a
Baronesse	1.14 ± 0.08	a	0.56 ± 0.07	a-c	3.56 ± 0.26	a	5.26 ± 0.42	a	0.47 ± 0.04	a	0.98 ± 0.04	a	2.80 ± 0.19	a	4.24 ± 0.27	a	5.82 ± 0.23	a	10.06 ± 0.49	a
Braemar	1.22 ± 0.09	a	0.49 ± 0.09	a-c	3.74 ± 0.33	a	5.44 ± 0.50	a	0.51 ± 0.05	a	0.90 ± 0.03	a	2.97 ± 0.38	a	4.38 ± 0.45	a	6.68 ± 0.48	a	11.05 ± 0.92	a
Carina	1.16 ± 0.16	a	0.33 ± 0.04	a-c	3.93 ± 0.39	a	5.43 ± 0.58	a	0.70 ± 0.18	a	0.85 ± 0.07	a	3.53 ± 0.44	a	5.08 ± 0.62	a	5.70 ± 0.45	a	10.78 ± 1.05	a
Grace	1.19 ± 0.10	a	0.42 ± 0.03	a-c	3.45 ± 0.17	a	5.06 ± 0.27	a	0.49 ± 0.07	a	0.88 ± 0.04	a	3.06 ± 0.31	a	4.44 ± 0.41	a	6.58 ± 0.50	a	11.03 ± 0.91	a
IPZ 24727	1.23 ± 0.21	a	0.59 ± 0.08	c	3.20 ± 0.73	a	5.02 ± 0.99	a	0.48 ± 0.11	a	1.05 ± 0.17	a	2.99 ± 0.65	a	4.52 ± 0.93	a	5.65 ± 0.73	a	10.17 ± 1.66	a
Irina	1.33 ± 0.03	a	0.35 ± 0.04	a-c	3.35 ± 0.21	a	5.03 ± 0.20	a	0.62 ± 0.05	a	0.91 ± 0.04	a	2.94 ± 0.16	a	4.46 ± 0.22	a	6.94 ± 0.33	a	11.40 ± 0.54	a
Mackay	1.22 ± 0.08	a	0.37 ± 0.04	a-c	3.26 ± 0.40	a	4.85 ± 0.47	a	0.52 ± 0.06	a	0.95 ± 0.08	a	2.83 ± 0.29	a	4.30 ± 0.42	a	6.25 ± 0.50	a	10.54 ± 0.90	a
Marthe	1.60 ± 0.12	a	0.40 ± 0.01	a-c	4.89 ± 0.36	a	6.90 ± 0.48	a	0.65 ± 0.09	a	1.24 ± 0.14	a	3.22 ± 0.33	a	5.12 ± 0.55	a	7.41 ± 0.64	a	12.53 ± 1.19	a
Melius	1.17 ± 0.15	a	0.41 ± 0.06	a-c	3.55 ± 0.54	a	5.14 ± 0.74	a	0.58 ± 0.12	a	0.88 ± 0.16	a	2.63 ± 0.33	a	4.08 ± 0.48	a	6.60 ± 0.56	a	10.68 ± 1.01	a
Power	1.35 ± 0.10	a	0.46 ± 0.07	a-c	3.59 ± 0.29	a	5.40 ± 0.45	a	0.58 ± 0.08	a	0.98 ± 0.11	a	2.65 ± 0.24	a	4.20 ± 0.43	a	6.98 ± 0.61	a	11.18 ± 1.03	a
Quench	1.27 ± 0.15	a	0.39 ± 0.05	a-c	3.65 ± 0.50	a	5.31 ± 0.65	a	0.52 ± 0.08	a	0.96 ± 0.11	a	2.79 ± 0.40	a	4.27 ± 0.57	a	6.67 ± 0.79	a	10.94 ± 1.35	a
Salome	1.13 ± 0.09	a	0.39 ± 0.07	a-c	4.07 ± 0.14	a	5.59 ± 0.30	a	0.51 ± 0.07	a	0.85 ± 0.12	a	2.70 ± 0.25	a	4.07 ± 0.43	a	7.16 ± 0.60	a	11.23 ± 1.03	a
Scarlett	1.23 ± 0.16	a	0.57 ± 0.04	bc	4.04 ± 0.49	a	5.84 ± 0.63	a	0.49 ± 0.06	a	1.18 ± 0.16	a	2.23 ± 0.52	a	3.90 ± 0.41	a	6.42 ± 0.43	a	10.31 ± 0.57	a
Shakira	1.01 ± 0.10	a	0.28 ± 0.01	a	3.22 ± 0.38	a	4.52 ± 0.47	a	0.45 ± 0.03	a	0.71 ± 0.06	a	2.50 ± 0.18	a	3.66 ± 0.20	a	5.14 ± 0.28	a	8.81 ± 0.43	a
Sissy	1.19 ± 0.05	a	0.43 ± 0.03	a-c	3.86 ± 0.21	a	5.47 ± 0.27	a	0.63 ± 0.12	a	0.81 ± 0.10	a	2.86 ± 0.17	a	4.30 ± 0.20	a	5.47 ± 0.08	a	9.77 ± 0.24	a
Solist	1.07 ± 0.10	a	0.40 ± 0.04	a-c	4.02 ± 0.53	a	5.50 ± 0.63	a	0.63 ± 0.16	a	0.93 ± 0.15	a	2.75 ± 0.35	a	4.31 ± 0.51	a	7.43 ± 0.73	a	11.73 ± 1.23	a
Trumpf	1.45 ± 0.09	a	0.38 ± 0.05	a-c	3.65 ± 0.39	a	5.48 ± 0.48	a	0.62 ± 0.07	a	0.91 ± 0.12	a	2.97 ± 0.21	a	4.50 ± 0.38	a	6.44 ± 0.50	a	10.94 ± 0.88	a
Union	1.41 ± 0.09	a	0.40 ± 0.03	a-c	4.24 ± 0.29	a	6.05 ± 0.35	a	0.84 ± 0.17	a	0.88 ± 0.04	a	3.95 ± 0.27	a	5.67 ± 0.41	a	7.05 ± 0.44	a	12.71 ± 0.81	a
Ursa	1.19 ± 0.05	a	0.39 ± 0.06	a-c	3.63 ± 0.14	a	5.21 ± 0.18	a	0.52 ± 0.05	a	0.98 ± 0.07	a	2.89 ± 0.20	a	4.39 ± 0.23	a	6.14 ± 0.25	a	10.53 ± 0.46	a
Volla	1.55 ± 0.17	a	0.44 ± 0.05	a-c	4.20 ± 0.43	a	6.19 ± 0.64	a	0.72 ± 0.11	a	0.99 ± 0.12	a	3.66 ± 0.39	a	5.38 ± 0.61	a	6.12 ± 0.37	a	11.50 ± 0.98	a
Wiebke	1.34 ± 0.09	a	0.48 ± 0.02	a-c	4.09 ± 0.31	a	5.91 ± 0.39	a	0.59 ± 0.05	a	0.99 ± 0.12	a	3.45 ± 0.21	a	5.03 ± 0.32	a	7.04 ± 0.34	a	12.06 ± 0.65	a
**Mean**	1.26		0.42		3.75		5.42		0.58		0.94		2.96		4.48		6.41		10.89	
**Min**	1.01		0.28		3.20		4.52		0.45		0.71		2.23		3.66		5.14		8.81	
**Max**	1.60		0.59		4.89		6.90		0.84		1.24		3.95		5.67		7.43		12.71	

Supplementary Table 2 Mean (± SE) and Tukey-test for dry matter (DM) translocation of leaves, sheaths, (stems+ears) and shoot total from pre-anthesis to grain weight at maturity, the grain DM of photosynthetic assimilates from post-anthesis, and total DM translocation efficiency (DMTE) (%) in 2014. Mean comparisons among varieties from Tukey´s HSD test indicate a significant difference at $p < 0.05$. The same letters mean groups in a column that were not significantly different from one another.

Variety Name	Translocation (t ha^{-1})				Post anthesis assimilates (t ha^{-1})	DMTE (%)	Contribution	
	Leaves	Sheaths	Stems+ears	Shoot total			Assimilation pre-anthesis	Assimilation post-anthesis
	mean±SE	mean±SE	mean±SE	mean±SE	mean±SE	mean±SE	mean±SE	mean±SE
Aspen	0.60 ± 0.04 ab	-0.54 ± 0.09 a	0.65 ± 0.19 a-c	0.71 ± 0.18 ab	4.98 ± 0.34 a	13.5 ± 3.10 ab	12 ± 0.39 ab	88 ± 2.8
Barke	0.66 ± 0.09 ab	-0.58 ± 0.13 a	0.57 ± 0.22 a-c	0.66 ± 0.23 ab	5.43 ± 0.68 a	12.7 ± 3.39 ab	11 ± 0.41 ab	89 ± 3.4
Baronesse	0.67 ± 0.06 ab	-0.41 ± 0.06 a	0.77 ± 0.25 a-c	1.02 ± 0.36 ab	4.79 ± 0.43 a	18.7 ± 5.36 ab	18 ± 1.27 ab	82 ± 5.9
Braemar	0.70 ± 0.04 ab	-0.41 ± 0.07 a	0.78 ± 0.07 a-c	1.07 ± 0.06 ab	5.61 ± 0.42 a	19.8 ± 0.94 ab	16 ± 0.09 ab	84 ± 0.5
Carina	0.47 ± 0.06 a	-0.52 ± 0.07 a	0.40 ± 0.07 a-c	0.35 ± 0.10 a	5.35 ± 0.52 a	6.9 ± 2.66 a	7 ± 0.18 a	93 ± 2.5
Grace	0.70 ± 0.04 ab	-0.46 ± 0.02 a	0.38 ± 0.15 a-c	0.62 ± 0.14 ab	5.97 ± 0.64 a	12.7 ± 3.51 ab	10 ± 0.32 ab	90 ± 2.9
IPZ 24727	0.75 ± 0.11 ab	-0.46 ± 0.12 a	0.21 ± 0.14 a	0.50 ± 0.11 ab	5.15 ± 0.70 a	10.4 ± 2.07 ab	9 ± 0.20 ab	91 ± 2.0
Irina	0.71 ± 0.04 ab	-0.56 ± 0.08 a	0.41 ± 0.08 a-c	0.56 ± 0.08 ab	6.38 ± 0.39 a	11.3 ± 1.81 ab	8 ± 0.15 ab	92 ± 1.6
Mackay	0.70 ± 0.03 ab	-0.58 ± 0.06 a	0.43 ± 0.23 a-c	0.56 ± 0.24 ab	5.69 ± 0.55 a	11.3 ± 4.17 ab	9 ± 0.37 ab	91 ± 3.7
Marthe	0.95 ± 0.04 b	-0.84 ± 0.14 a	1.67 ± 0.06 bc	1.78 ± 0.08 ab	5.64 ± 0.71 a	26.4 ± 3.23 ab	25 ± 1.17 ab	75 ± 3.5
Melius	0.60 ± 0.16 ab	-0.47 ± 0.13 a	0.93 ± 0.30 a-c	1.06 ± 0.38 ab	5.54 ± 0.39 a	19.3 ± 4.46 ab	15 ± 0.83 ab	85 ± 4.6
Power	0.77 ± 0.03 ab	-0.52 ± 0.06 a	0.94 ± 0.11 a-c	1.20 ± 0.17 ab	5.78 ± 0.63 a	22.5 ± 3.24 ab	18 ± 0.61 ab	82 ± 2.9
Quench	0.75 ± 0.08 ab	-0.57 ± 0.11 a	0.86 ± 0.26 a-c	1.05 ± 0.19 ab	5.63 ± 0.73 a	19.8 ± 2.98 ab	16 ± 0.53 ab	84 ± 2.8
Salome	0.62 ± 0.02 ab	-0.47 ± 0.08 a	1.36 ± 0.11 a-c	1.52 ± 0.16 ab	5.64 ± 0.75 a	27.8 ± 4.24 b	22 ± 1.17 ab	78 ± 4.1
Scarlett	0.74 ± 0.11 ab	-0.61 ± 0.18 a	1.81 ± 0.92 c	1.94 ± 0.83 b	4.48 ± 0.56 a	30.6 ± 9.76 ab	29 ± 4.22 b	71 ± 10.5
Shakira	0.56 ± 0.08 ab	-0.43 ± 0.07 a	0.72 ± 0.21 a-c	0.85 ± 0.31 ab	4.29 ± 0.06 a	17.4 ± 4.96 ab	16 ± 0.93 ab	84 ± 5.0
Sissy	0.56 ± 0.07 ab	-0.38 ± 0.11 a	1.00 ± 0.24 a-c	1.17 ± 0.31 ab	4.30 ± 0.26 a	20.9 ± 5.27 ab	21 ± 1.50 ab	79 ± 5.6
Solist	0.45 ± 0.17 a	-0.53 ± 0.13 a	1.28 ± 0.32 a-c	1.19 ± 0.35 ab	6.24 ± 0.71 a	21.3 ± 4.60 ab	16 ± 0.79 ab	84 ± 4.1
Trumpf	0.83 ± 0.04 ab	-0.53 ± 0.09 a	0.67 ± 0.18 a-c	0.98 ± 0.14 ab	5.47 ± 0.42 a	17.7 ± 1.47 ab	15 ± 0.27 ab	85 ± 1.5
Union	0.58 ± 0.12 ab	-0.49 ± 0.04 a	0.29 ± 0.02 ab	0.38 ± 0.10 a	6.66 ± 0.49 a	6.5 ± 1.88 a	6 ± 0.10 a	94 ± 1.6
Ursa	0.67 ± 0.05 ab	-0.58 ± 0.06 a	0.74 ± 0.26 a-c	0.82 ± 0.26 ab	5.32 ± 0.46 a	15.6 ± 4.99 ab	14 ± 0.73 ab	86 ± 4.5
Volla	0.82 ± 0.06 ab	-0.55 ± 0.09 a	0.54 ± 0.21 a-c	0.81 ± 0.23 ab	5.31 ± 0.48 a	13.1 ± 3.90 ab	13 ± 0.68 ab	87 ± 4.4
Wiebke	0.76 ± 0.05 ab	-0.51 ± 0.13 a	0.64 ± 0.19 a-c	0.89 ± 0.10 ab	6.15 ± 0.28 a	15.0 ± 1.15 ab	13 ± 0.15 ab	87 ± 1.1
**Mean**	0.68	-0.52	0.78	0.94	5.47	17.01	15	85
**Min**	0.45	-0.84	0.21	0.35	4.29	6.53	6	71
**Max**	0.95	-0.38	1.81	1.94	6.66	30.64	29	94

Supplementary Table 3 Dry matter (DM) partitioning of 23 spring malting barley varieties under the optimal N fertilization in 2015. Mean (± SE) and Tukey-test for (DM) accumulation of leaves, sheaths, (stems+ears) or (stems+chaff) at anthesis and at maturity, and grain and total aboveground DM of spring malting barley varieties. Mean comparisons among varieties from Tukey´s HSD test indicate a significant difference at $p < 0.05$. The same letters mean groups in a column that were not significantly different from one another.

Variety Name	Dry matter partitioning (t ha^{-1})									
	Anthesis				Maturity					
	Leaves	Sheaths	Stems+Ears	Shoot total	Leaves	Sheaths	Stems+Chaff	Straw	Grain	Aboveground organs
	mean±SE	mean±SE	mean±SE	mean±SE	mean±SE	mean±SE	mean±SE	mean±SE	mean±SE	mean±SE
Aspen	1.00 ± 0.19 a	0.64 ± 0.06 ab	4.85 ± 0.77 ab	6.50 ± 1.01 a	0.58 ± 0.08 a	0.94 ± 0.17 a	3.06 ± 0.48 ab	4.59 ± 0.73 ab	6.20 ± 0.57 a	10.79 ± 1.29 a
Barke	0.78 ± 0.11 a	0.53 ± 0.07 ab	3.94 ± 0.40 ab	5.25 ± 0.56 a	0.43 ± 0.04 a	0.82 ± 0.09 a	2.65 ± 0.22 ab	3.90 ± 0.35 ab	6.02 ± 0.53 a	9.91 ± 0.88 a
Baronesse	0.86 ± 0.08 a	0.82 ± 0.05 ab	5.64 ± 0.61 ab	7.32 ± 0.73 a	0.53 ± 0.09 a	1.07 ± 0.15 a	3.10 ± 0.52 ab	4.70 ± 0.76 ab	7.58 ± 0.94 a	12.28 ± 1.70 a
Braemar	0.83 ± 0.11 a	0.75 ± 0.03 ab	4.88 ± 0.39 ab	6.45 ± 0.51 a	0.50 ± 0.02 a	0.90 ± 0.12 a	3.11 ± 0.13 ab	4.50 ± 0.24 ab	6.93 ± 0.28 a	11.44 ± 0.48 a
Carina	1.18 ± 0.17 a	0.62 ± 0.05 ab	6.25 ± 0.60 b	8.05 ± 0.81 a	0.65 ± 0.08 a	1.22 ± 0.17 a	4.06 ± 0.32 b	5.93 ± 0.54 b	7.42 ± 0.86 a	13.35 ± 1.38 a
Grace	0.74 ± 0.06 a	0.62 ± 0.00 ab	4.48 ± 0.26 ab	5.83 ± 0.31 a	0.46 ± 0.04 a	0.85 ± 0.06 a	2.69 ± 0.23 ab	4.00 ± 0.26 ab	6.97 ± 0.26 a	10.97 ± 0.47 a
IPZ 24727	0.93 ± 0.16 a	0.93 ± 0.11 b	5.13 ± 0.37 ab	6.99 ± 0.63 a	0.55 ± 0.06 a	1.17 ± 0.08 a	3.57 ± 0.20 ab	5.29 ± 0.32 ab	6.91 ± 0.47 a	12.20 ± 0.70 a
Irina	1.09 ± 0.08 a	0.65 ± 0.02 ab	4.91 ± 0.32 ab	6.65 ± 0.41 a	0.57 ± 0.05 a	0.86 ± 0.12 a	3.19 ± 0.24 ab	4.62 ± 0.31 ab	7.14 ± 0.40 a	11.76 ± 0.71 a
Mackay	0.88 ± 0.17 a	0.80 ± 0.09 ab	4.53 ± 0.90 ab	6.21 ± 1.16 a	0.50 ± 0.12 a	0.80 ± 0.22 a	2.67 ± 0.40 ab	3.97 ± 0.72 ab	6.25 ± 1.20 a	10.22 ± 1.92 a
Marthe	1.00 ± 0.04 a	0.73 ± 0.05 ab	5.04 ± 0.07 ab	6.78 ± 0.10 a	0.58 ± 0.04 a	1.11 ± 0.05 a	2.95 ± 0.12 ab	4.63 ± 0.19 ab	7.02 ± 0.21 a	11.65 ± 0.36 a
Melius	0.94 ± 0.06 a	0.69 ± 0.07 ab	4.75 ± 0.16 ab	6.37 ± 0.23 a	0.51 ± 0.06 a	0.81 ± 0.21 a	2.87 ± 0.07 ab	4.19 ± 0.29 ab	6.88 ± 0.43 a	11.07 ± 0.71 a
Power	0.79 ± 0.05 a	0.52 ± 0.06 a	3.55 ± 0.16 a	4.86 ± 0.26 a	0.47 ± 0.06 a	0.64 ± 0.12 a	2.63 ± 0.28 ab	3.74 ± 0.35 ab	6.13 ± 0.43 a	9.86 ± 0.74 a
Quench	0.98 ± 0.14 a	0.76 ± 0.12 ab	4.59 ± 0.56 ab	6.33 ± 0.81 a	0.60 ± 0.11 a	0.90 ± 0.13 a	3.01 ± 0.30 ab	4.51 ± 0.50 ab	6.60 ± 0.74 a	11.11 ± 1.23 a
Salome	0.85 ± 0.14 a	0.87 ± 0.07 ab	4.59 ± 0.58 ab	6.31 ± 0.79 a	0.42 ± 0.05 a	0.83 ± 0.09 a	2.24 ± 0.35 a	3.50 ± 0.49 a	5.92 ± 0.51 a	9.42 ± 1.00 a
Scarlett	0.74 ± 0.10 a	0.77 ± 0.11 ab	4.09 ± 0.42 ab	5.60 ± 0.61 a	0.38 ± 0.05 a	0.88 ± 0.11 a	2.37 ± 0.32 a	3.63 ± 0.47 ab	6.32 ± 0.81 a	9.95 ± 1.27 a
Shakira	1.07 ± 0.12 a	0.53 ± 0.06 ab	5.50 ± 0.63 ab	7.10 ± 0.80 a	0.69 ± 0.10 a	0.91 ± 0.19 a	3.35 ± 0.36 ab	4.95 ± 0.59 ab	7.05 ± 0.90 a	11.99 ± 1.48 a
Sissy	1.09 ± 0.25 a	0.68 ± 0.13 ab	5.61 ± 0.75 ab	7.38 ± 1.11 a	0.55 ± 0.08 a	0.96 ± 0.22 a	3.61 ± 0.24 ab	5.12 ± 0.52 ab	6.31 ± 0.76 a	11.43 ± 1.28 a
Solist	0.93 ± 0.06 a	0.68 ± 0.04 ab	5.07 ± 0.30 ab	6.68 ± 0.39 a	0.49 ± 0.05 a	0.95 ± 0.10 a	3.05 ± 0.17 ab	4.49 ± 0.28 ab	7.29 ± 0.50 a	11.79 ± 0.76 a
Trumpf	1.01 ± 0.11 a	0.69 ± 0.07 ab	4.29 ± 0.37 ab	5.99 ± 0.55 a	0.53 ± 0.02 a	0.81 ± 0.05 a	2.89 ± 0.11 ab	4.23 ± 0.17 ab	5.99 ± 0.24 a	10.23 ± 0.40 a
Union	1.02 ± 0.05 a	0.87 ± 0.04 ab	5.12 ± 0.19 ab	7.01 ± 0.27 a	0.52 ± 0.06 a	0.90 ± 0.11 a	3.06 ± 0.29 ab	4.48 ± 0.45 ab	6.40 ± 0.53 a	10.88 ± 0.95 a
Ursa	0.91 ± 0.05 a	0.62 ± 0.03 ab	5.18 ± 0.29 ab	6.71 ± 0.36 a	0.50 ± 0.03 a	0.93 ± 0.19 a	3.41 ± 0.15 ab	4.85 ± 0.08 ab	6.57 ± 0.46 a	11.42 ± 0.44 a
Volla	0.95 ± 0.08 a	0.71 ± 0.06 ab	4.77 ± 0.16 ab	6.43 ± 0.28 a	0.53 ± 0.04 a	1.02 ± 0.04 a	3.55 ± 0.16 ab	5.10 ± 0.23 ab	6.31 ± 0.22 a	11.41 ± 0.44 a
Wiebke	0.90 ± 0.14 a	0.73 ± 0.16 ab	4.71 ± 0.40 ab	6.35 ± 0.67 a	0.46 ± 0.06 a	0.85 ± 0.03 a	3.22 ± 0.40 ab	4.53 ± 0.47 ab	6.29 ± 0.54 a	10.82 ± 1.01 a
**Mean**	0.93	0.70	4.85	6.49	0.52	0.92	3.06	4.50	6.63	11.13
**Min**	0.74	0.52	3.55	4.86	0.38	0.64	2.24	3.50	5.92	9.42
**Max**	1.18	0.93	6.25	8.05	0.69	1.22	4.06	5.93	7.58	13.35

Supplementary Table 4 Mean (± SE) and Tukey-test for dry matter (DM) translocation of leaves, sheaths, (stems+ears) and shoot total from pre-anthesis to grain weight at maturity, the grain DM of photosynthetic assimilates from post-anthesis, and total DM translocation efficiency (DMTE) (%) in 2015. Mean comparisons among varieties from Tukey´s HSD test indicate a significant difference at $p < 0.05$. The same letters mean groups in a column that were not significantly different from one another.

Variety Name	Translocation ($t\ ha^{-1}$)								Post anthesis assimilates ($t\ ha^{-1}$)		DMTE (%)		Contribution			
	Leaves		Sheaths		Stems+ears		Shoot total						Assimilation pre-anthesis		Assimilation post-anthesis	
	mean±SE		mean±SE		mean±SE		mean±SE		mean±SE		mean±SE		mean±SE		mean±SE	
Aspen	0.42 ± 0.11	a	-0.30 ± 0.11	ab	1.79 ± 0.34	ab	1.91 ± 0.38	a	4.29 ± 0.40	a	29.0 ± 4.0	ab	30 ± 2.3	ab	70 ± 5.3	ab
Barke	0.35 ± 0.07	a	-0.28 ± 0.09	ab	1.29 ± 0.22	ab	1.35 ± 0.29	a	4.66 ± 0.40	a	25.1 ± 3.5	ab	22 ± 1.1	ab	78 ± 3.7	ab
Baronesse	0.33 ± 0.03	a	-0.26 ± 0.10	ab	2.54 ± 0.14	b	2.62 ± 0.12	a	4.96 ± 0.99	a	36.8 ± 3.7	ab	36 ± 2.4	ab	64 ± 4.3	ab
Braemar	0.33 ± 0.11	a	-0.16 ± 0.09	ab	1.77 ± 0.28	ab	1.95 ± 0.35	a	4.99 ± 0.14	a	29.7 ± 3.3	ab	28 ± 1.5	ab	72 ± 3.8	ab
Carina	0.53 ± 0.10	a	-0.60 ± 0.13	a	2.19 ± 0.28	ab	2.12 ± 0.28	a	5.31 ± 0.68	a	26.0 ± 1.3	ab	29 ± 1.0	ab	71 ± 2.6	ab
Grace	0.28 ± 0.02	a	-0.23 ± 0.07	ab	1.79 ± 0.16	ab	1.83 ± 0.10	a	5.14 ± 0.24	a	31.5 ± 1.4	ab	26 ± 0.5	ab	74 ± 1.3	ab
IPZ 24727	0.37 ± 0.12	a	-0.24 ± 0.04	ab	1.57 ± 0.36	ab	1.70 ± 0.52	a	5.21 ± 0.33	a	23.3 ± 5.2	a	24 ± 1.8	a	76 ± 5.8	a
Irina	0.52 ± 0.04	a	-0.20 ± 0.11	ab	1.72 ± 0.28	ab	2.03 ± 0.21	a	5.11 ± 0.37	a	30.5 ± 2.7	ab	29 ± 1.2	ab	71 ± 3.0	ab
Mackay	0.38 ± 0.08	a	0.00 ± 0.14	ab	1.86 ± 0.55	ab	2.24 ± 0.48	a	4.01 ± 0.79	a	35.4 ± 3.5	ab	35 ± 2.3	ab	65 ± 4.2	ab
Marthe	0.42 ± 0.04	a	-0.37 ± 0.02	ab	2.09 ± 0.13	ab	2.14 ± 0.15	a	4.88 ± 0.36	a	31.6 ± 2.3	ab	31 ± 1.4	ab	69 ± 3.1	ab
Melius	0.42 ± 0.02	a	-0.12 ± 0.18	ab	1.88 ± 0.19	ab	2.18 ± 0.11	a	4.70 ± 0.49	a	34.5 ± 2.5	ab	32 ± 1.5	ab	68 ± 3.2	ab
Power	0.32 ± 0.04	a	-0.12 ± 0.15	ab	0.93 ± 0.17	a	1.12 ± 0.24	a	5.00 ± 0.59	a	23.3 ± 5.0	a	19 ± 1.0	ab	81 ± 4.4	ab
Quench	0.37 ± 0.10	a	-0.14 ± 0.08	ab	1.58 ± 0.27	ab	1.82 ± 0.39	a	4.78 ± 0.48	a	28.1 ± 2.9	ab	27 ± 1.2	ab	73 ± 3.3	ab
Salome	0.42 ± 0.10	a	0.03 ± 0.03	a	2.35 ± 0.26	ab	2.81 ± 0.34	a	3.11 ± 0.34	a	44.8 ± 2.3	b	47 ± 3.2	b	53 ± 3.6	b
Scarlett	0.37 ± 0.06	a	-0.12 ± 0.02	ab	1.72 ± 0.20	ab	1.97 ± 0.25	a	4.35 ± 0.73	a	35.6 ± 3.3	ab	32 ± 2.0	ab	68 ± 4.2	ab
Shakira	0.38 ± 0.03	a	-0.37 ± 0.13	ab	2.15 ± 0.35	ab	2.16 ± 0.27	a	4.89 ± 0.70	a	30.5 ± 2.0	ab	31 ± 1.2	ab	69 ± 2.6	ab
Sissy	0.54 ± 0.19	a	-0.27 ± 0.20	ab	1.99 ± 0.58	ab	2.26 ± 0.76	a	4.05 ± 0.76	a	28.9 ± 5.5	ab	35 ± 5.0	ab	65 ± 9.4	ab
Solist	0.44 ± 0.04	a	-0.27 ± 0.08	ab	2.02 ± 0.21	ab	2.19 ± 0.31	a	5.11 ± 0.59	a	32.5 ± 3.8	ab	30 ± 2.1	ab	70 ± 4.8	ab
Trumpf	0.49 ± 0.11	a	-0.12 ± 0.05	ab	1.40 ± 0.28	ab	1.76 ± 0.42	a	4.23 ± 0.45	a	28.3 ± 4.2	ab	29 ± 2.9	ab	71 ± 7.0	ab
Union	0.50 ± 0.06	a	-0.03 ± 0.13	ab	2.06 ± 0.25	ab	2.53 ± 0.44	a	3.87 ± 0.82	a	36.1 ± 6.1	ab	41 ± 6.7	ab	59 ± 9.5	ab
Ursa	0.41 ± 0.05	a	-0.31 ± 0.19	ab	1.76 ± 0.34	ab	1.87 ± 0.42	a	4.70 ± 0.23	a	27.0 ± 5.1	ab	28 ± 2.0	ab	72 ± 5.3	ab
Volla	0.42 ± 0.08	a	-0.31 ± 0.06	ab	1.22 ± 0.18	ab	1.33 ± 0.31	a	4.98 ± 0.40	a	20.4 ± 4.0	a	21 ± 1.3	a	79 ± 4.9	a
Wiebke	0.44 ± 0.08	a	-0.12 ± 0.15	ab	1.49 ± 0.12	ab	1.81 ± 0.32	a	4.47 ± 0.45	a	28.4 ± 2.9	ab	29 ± 1.5	ab	71 ± 3.7	ab
**Mean**	0.41		-0.21		1.79		1.99		###		30.30		30		70	
**Min**	0.28		-0.60		0.93		1.12		###		20.44		19		53	
**Max**	0.54		0.03		2.54		2.81		###		44.76		47		81	

Supplementary Table 5 Nitrogen (N) partitioning of 23 spring malting barley varieties under the optimal N fertilization in 2014. Mean (± SE) and Tukey-test for (N) accumulation of leaves, sheaths, (stems+ears) or (stems+chaff) at anthesis and at maturity, and grain and total aboveground N of spring malting barley varieties. Mean comparisons among varieties from Tukey´s HSD test indicate a significant difference at $p < 0.05$. The same letters mean groups in a column that were not significantly different from one another.

Variety Name	Nitrogen partitioning (kg ha^{-1})									
	Anthesis				Maturity					
	Leaves	Sheaths	Stems+Ears	Shoot total	Leaves	Sheaths	Stems+Chaff	Straw	Grain	Aboveground organs
	mean±SE	mean±SE	mean±SE	mean±SE	mean±SE	mean±SE	mean±SE	mean±SE	mean±SE	mean±SE
Aspen	26.8 ± 0.9 a	2.6 ± 0.2 a	44.8 ± 2.7 ab	74.2 ± 3.4 a	3.2 ± 0.4 a	2.3 ± 0.3 ab	8.7 ± 0.9 a	14.2 ± 1.5 a	79.5 ± 8.6 a	93.7 ± 10.0 a
Barke	30.3 ± 4.6 a	4.4 ± 0.6 ab	47.2 ± 7.0 ab	81.9 ± 12.1 a	2.9 ± 0.6 a	2.6 ± 0.4 ab	7.3 ± 0.7 a	12.8 ± 1.7 a	82.7 ± 11.3 a	95.5 ± 13.0 a
Baronesse	24.4 ± 1.7 a	4.6 ± 0.5 ab	42.4 ± 3.1 ab	71.3 ± 5.2 a	2.1 ± 0.2 a	2.6 ± 0.1 ab	7.2 ± 0.5 a	11.8 ± 0.7 a	80.7 ± 4.3 a	92.6 ± 5.0 a
Braemar	28.1 ± 2.9 a	4.0 ± 0.8 ab	49.9 ± 3.6 ab	82.0 ± 7.2 a	2.3 ± 0.3 a	2.5 ± 0.2 ab	8.4 ± 1.4 a	13.2 ± 1.8 a	84.7 ± 7.2 a	97.9 ± 9.0 a
Carina	24.5 ± 4.0 a	3.0 ± 0.4 a	44.0 ± 4.6 ab	71.5 ± 8.9 a	2.4 ± 0.3 a	2.6 ± 0.4 ab	7.4 ± 1.2 a	12.3 ± 1.9 a	76.6 ± 7.3 a	88.9 ± 8.9 a
Grace	29.7 ± 4.8 a	3.6 ± 0.5 a	43.9 ± 3.8 ab	77.2 ± 8.9 a	2.5 ± 0.6 a	2.2 ± 0.2 ab	8.3 ± 1.1 a	13.0 ± 1.9 a	90.3 ± 10.5 a	103.3 ± 12.4 a
IPZ 24727	33.4 ± 7.7 a	4.9 ± 0.7 ab	52.7 ± 11.1 ab	90.9 ± 19.2 a	2.5 ± 0.8 a	3.0 ± 0.6 ab	9.4 ± 2.5 a	14.9 ± 3.9 a	88.3 ± 14.6 a	103.2 ± 18.4 a
Irina	30.2 ± 1.3 a	2.9 ± 0.2 a	43.5 ± 2.4 ab	76.7 ± 3.4 a	3.1 ± 0.3 a	2.3 ± 0.1 ab	8.6 ± 0.5 a	14.0 ± 0.8 a	85.8 ± 5.0 a	99.7 ± 5.8 a
Mackay	28.3 ± 3.2 a	3.3 ± 0.5 a	40.4 ± 3.9 ab	71.9 ± 7.3 a	2.8 ± 0.5 a	2.6 ± 0.2 ab	8.5 ± 1.2 a	13.9 ± 1.7 a	80.1 ± 7.7 a	94.0 ± 9.2 a
Marthe	40.1 ± 4.6 a	4.3 ± 0.3 ab	67.0 ± 6.6 b	111.3 ± 11.4 a	3.7 ± 0.5 a	4.1 ± 0.3 b	9.3 ± 0.4 a	17.1 ± 0.9 a	104.3 ± 13.4 a	121.4 ± 14.1 a
Melius	31.2 ± 3.1 a	4.4 ± 0.5 ab	45.4 ± 6.7 ab	81.0 ± 9.7 a	2.4 ± 0.4 a	2.6 ± 0.2 ab	8.2 ± 1.3 a	13.2 ± 1.9 a	79.3 ± 8.7 a	92.5 ± 10.4 a
Power	32.2 ± 3.4 a	4.1 ± 0.7 ab	47.7 ± 4.6 ab	84.0 ± 8.6 a	2.7 ± 0.5 a	2.4 ± 0.3 ab	7.0 ± 0.8 a	12.1 ± 1.5 a	85.9 ± 8.6 a	98.0 ± 10.1 a
Quench	29.4 ± 5.0 a	3.6 ± 0.8 a	48.1 ± 7.1 ab	81.1 ± 12.5 a	2.4 ± 0.5 a	2.3 ± 0.3 ab	7.6 ± 1.4 a	12.3 ± 2.1 a	84.3 ± 13.3 a	96.6 ± 15.4 a
Salome	27.6 ± 3.3 a	3.6 ± 0.8 a	51.0 ± 2.3 ab	82.2 ± 5.7 a	3.0 ± 1.0 a	2.3 ± 0.5 ab	7.3 ± 0.5 a	12.6 ± 1.9 a	83.9 ± 8.3 a	96.5 ± 10.0 a
Scarlett	31.6 ± 4.6 a	5.1 ± 0.2 ab	52.6 ± 6.5 ab	89.2 ± 10.9 a	2.5 ± 0.4 a	3.2 ± 0.9 ab	11.0 ± 3.7 a	16.7 ± 4.9 a	87.1 ± 9.6 a	103.8 ± 14.4 a
Shakira	24.1 ± 2.1 a	2.3 ± 0.1 a	37.0 ± 3.4 a	63.4 ± 5.5 a	2.3 ± 0.2 a	1.9 ± 0.3 a	7.4 ± 0.6 a	11.6 ± 0.9 a	63.2 ± 2.4 a	74.8 ± 2.8 a
Sissy	25.6 ± 0.8 a	3.7 ± 0.2 ab	44.6 ± 2.2 ab	74.0 ± 2.9 a	2.2 ± 0.1 a	2.2 ± 0.0 ab	7.7 ± 0.9 a	12.2 ± 1.0 a	75.6 ± 1.9 a	87.8 ± 2.7 a
Solist	27.5 ± 4.3 a	3.7 ± 0.4 ab	55.4 ± 7.4 ab	86.5 ± 11.8 a	2.7 ± 0.6 a	2.8 ± 0.3 ab	7.9 ± 1.7 a	13.3 ± 2.5 a	91.6 ± 12.6 a	105.0 ± 15.1 a
Trumpf	34.0 ± 3.0 a	3.3 ± 0.5 a	49.1 ± 4.7 ab	86.4 ± 7.7 a	3.0 ± 0.4 a	2.2 ± 0.2 ab	8.2 ± 0.6 a	13.4 ± 1.3 a	83.1 ± 6.6 a	96.4 ± 7.8 a
Union	34.7 ± 3.7 a	4.2 ± 0.6 ab	54.6 ± 5.2 ab	93.6 ± 8.9 a	3.9 ± 0.7 a	2.9 ± 0.5 ab	12.8 ± 1.5 a	19.7 ± 2.3 a	110.8 ± 10.7 a	130.5 ± 12.9 a
Ursa	30.5 ± 1.8 a	3.8 ± 0.4 ab	50.7 ± 0.8 ab	85.0 ± 2.1 a	2.6 ± 0.5 a	2.5 ± 0.2 ab	8.6 ± 0.7 a	13.8 ± 1.2 a	91.9 ± 5.8 a	105.7 ± 6.9 a
Volla	36.2 ± 4.8 a	4.2 ± 0.5 ab	54.6 ± 7.3 ab	94.9 ± 12.5 a	3.6 ± 0.7 a	2.6 ± 0.3 ab	11.6 ± 3.1 a	17.7 ± 4.0 a	86.9 ± 7.4 a	104.6 ± 11.4 a
Wiebke	32.0 ± 3.5 a	6.8 ± 1.5 b	53.3 ± 3.3 ab	92.1 ± 6.0 a	2.8 ± 0.2 a	2.5 ± 0.3 ab	9.2 ± 0.6 a	14.5 ± 1.1 a	95.9 ± 6.4 a	110.4 ± 7.5 a
**Mean**	30.1	3.9	48.7	82.7	2.7	2.6	8.6	13.9	85.8	99.7
**Min**	24.1	2.3	37.0	63.4	2.1	1.9	7.0	11.6	63.2	74.8
**Max**	40.1	6.8	67.0	111.3	3.9	4.1	12.8	19.7	110.8	130.5

Supplementary Table 6 Mean (± SE) and Tukey-test for nitrogen (N) translocation of leaves, sheaths, (stems+ears) and shoot total from pre-anthesis to grain weight at maturity, the grain N uptake from post-anthesis, and total N translocation efficiency (NTE) (%) in 2014. Mean comparisons among varieties from Tukey´s HSD test indicate a significant difference at $p < 0.05$. The same letters mean groups in a column that were not significantly different from one another.

Variety Name	Translocation (kg ha^{-1})								Post-anthesis N uptake (kg ha^{-1})		NTE (%)		Contribution			
	Leaves		Sheaths		Stems+ears		Shoot total						Assimilation pre-anthesis		Assimilation post-anthesis	
	mean±SE		mean±SE		mean±SE		mean±SE		mean±SE		mean±SE		mean±SE		mean±SE	
Aspen	23.6 ± 0.6	a	0.3 ± 0.4	a	36.1 ± 2.3	ab	60.0 ± 2.5	ab	19.5 ± 7.4	ab	81.0 ± 1.5	a	77.5 ± 6.7	a	22.5 ± 2.0	a
Barke	27.4 ± 4.1	a	1.7 ± 0.2	ab	39.9 ± 6.4	ab	69.0 ± 10.6	ab	13.6 ± 4.3	ab	83.9 ± 1.3	a	82.8 ± 5.5	a	17.2 ± 1.1	a
Baronesse	22.3 ± 1.6	a	1.9 ± 0.5	ab	35.3 ± 3.0	ab	59.5 ± 5.0	ab	21.2 ± 7.1	ab	83.3 ± 1.2	a	74.4 ± 7.7	a	25.6 ± 2.6	a
Braemar	25.8 ± 2.6	a	1.5 ± 0.7	ab	41.5 ± 2.4	ab	68.9 ± 5.6	ab	15.8 ± 3.2	ab	84.1 ± 1.0	a	81.5 ± 3.2	a	18.5 ± 0.7	a
Carina	22.1 ± 3.7	a	0.5 ± 0.1	a	36.6 ± 3.7	ab	59.2 ± 7.2	ab	17.4 ± 0.5	ab	82.9 ± 0.9	a	76.6 ± 2.5	a	23.4 ± 0.8	a
Grace	27.2 ± 4.2	a	1.4 ± 0.3	ab	35.6 ± 2.8	ab	64.2 ± 7.0	ab	26.1 ± 4.4	ab	83.3 ± 0.6	a	71.3 ± 2.7	a	28.7 ± 1.1	a
IPZ 24727	30.9 ± 7.0	a	1.9 ± 0.6	ab	43.3 ± 8.7	ab	76.0 ± 15.4	ab	12.2 ± 5.1	ab	84.1 ± 1.2	a	85.4 ± 5.0	a	14.6 ± 0.8	a
Irina	27.1 ± 1.2	a	0.7 ± 0.2	ab	34.9 ± 1.9	ab	62.7 ± 2.6	ab	23.0 ± 3.7	ab	81.8 ± 0.4	a	73.6 ± 3.1	a	26.4 ± 1.1	a
Mackay	25.5 ± 2.8	a	0.7 ± 0.4	ab	31.9 ± 3.0	a	58.1 ± 6.0	ab	22.1 ± 2.7	ab	80.7 ± 1.3	a	72.5 ± 2.1	a	27.5 ± 0.8	a
Marthe	36.4 ± 4.2	a	0.2 ± 0.1	a	57.7 ± 6.6	b	94.3 ± 10.7	b	10.0 ± 2.9	a	84.4 ± 1.3	a	91.1 ± 2.0	a	8.9 ± 0.2	a
Melius	28.8 ± 2.9	a	1.9 ± 0.4	ab	37.2 ± 5.7	ab	67.9 ± 8.3	ab	11.5 ± 3.8	a	83.8 ± 1.5	a	85.7 ± 4.4	a	14.3 ± 0.7	a
Power	29.5 ± 3.0	a	1.6 ± 0.5	ab	40.7 ± 3.8	ab	71.9 ± 7.3	ab	13.9 ± 4.1	ab	85.7 ± 0.7	a	84.1 ± 3.7	a	15.9 ± 0.7	a
Quench	27.0 ± 4.5	a	1.3 ± 0.6	a	40.4 ± 5.9	ab	68.8 ± 10.5	ab	15.6 ± 4.0	ab	84.9 ± 0.7	a	81.6 ± 3.5	a	18.4 ± 0.8	a
Salome	24.6 ± 2.5	a	1.3 ± 0.5	a	43.7 ± 1.9	ab	69.6 ± 4.0	ab	14.3 ± 4.9	ab	84.9 ± 1.3	a	84.3 ± 5.1	a	15.7 ± 0.9	a
Scarlett	29.1 ± 4.3	a	1.8 ± 0.9	ab	41.6 ± 3.9	ab	72.5 ± 7.8	ab	14.6 ± 7.7	ab	81.9 ± 3.5	a	84.1 ± 7.4	a	15.9 ± 1.4	a
Shakira	21.8 ± 2.0	a	0.4 ± 0.4	a	29.6 ± 3.0	a	51.8 ± 5.0	a	11.5 ± 3.1	a	81.6 ± 1.2	a	81.5 ± 5.2	a	18.5 ± 1.2	a
Sissy	23.5 ± 0.8	a	1.5 ± 0.2	ab	36.9 ± 2.3	ab	61.8 ± 3.0	ab	13.8 ± 3.0	ab	83.5 ± 1.5	a	81.8 ± 3.9	a	18.2 ± 0.9	a
Solist	24.8 ± 3.8	a	0.9 ± 0.5	a	47.5 ± 6.1	ab	73.2 ± 9.7	ab	18.5 ± 6.1	ab	84.8 ± 1.3	a	80.5 ± 5.0	a	19.5 ± 1.2	a
Trumpf	31.0 ± 2.6	a	1.1 ± 0.3	ab	40.9 ± 4.2	ab	73.0 ± 6.7	ab	10.0 ± 3.9	a	84.5 ± 0.8	a	88.0 ± 4.3	a	12.0 ± 0.6	a
Union	30.7 ± 3.6	a	1.3 ± 0.7	a	41.8 ± 4.5	ab	73.8 ± 7.5	ab	37.0 ± 8.5	b	78.9 ± 2.0	a	67.4 ± 5.8	a	32.6 ± 2.8	a
Ursa	27.9 ± 1.4	a	1.3 ± 0.4	ab	42.1 ± 1.1	ab	71.3 ± 1.2	ab	20.6 ± 5.2	ab	83.9 ± 1.0	a	78.3 ± 4.0	a	21.7 ± 1.1	a
Volla	32.6 ± 4.2	a	1.6 ± 0.3	ab	43.0 ± 4.4	ab	77.2 ± 8.6	ab	9.7 ± 2.7	a	81.9 ± 1.8	a	88.5 ± 3.4	a	11.5 ± 0.4	a
Wiebke	29.2 ± 3.3	a	4.3 ± 1.6	b	44.1 ± 2.8	ab	77.6 ± 5.0	ab	18.3 ± 3.0	ab	84.3 ± 0.5	a	81.1 ± 2.7	a	18.9 ± 0.6	a
**Mean**	27.4		1.3		40.1		68.8		17.0		83.2		80.6		19.4	
**Min**	21.8		0.2		29.6		51.8		9.7		78.9		67.4		8.9	
**Max**	36.4		4.3		57.7		94.3		37.0		85.7		91.1		32.6	

Supplementary Table 7 Nitrogen (N) partitioning of 23 spring malting barley varieties under the optimal N fertilization in 2014. Mean (± SE) and Tukey-test for (N) accumulation of leaves, sheaths, (stems+ears) or (stems+chaff) at anthesis and at maturity, and grain and total aboveground N of spring malting barley varieties. Mean comparisons among varieties from Tukey´s HSD test indicate a significant difference at $p < 0.05$. The same letters mean groups in a column that were not significantly different from one another.

Variety Name	Nitrogen partitioning (kg ha^{-1})																			
	Anthesis								Maturity											
	Leaves		Sheaths		Stems+Ears		Shoot total		Leaves		Sheaths		Stems+Chaff		Straw		Grain		Aboveground organs	
	mean±SE		mean±SE		mean±SE		mean±SE		mean±SE		mean±SE		mean±SE		mean±SE		mean±SE		mean±SE	
Aspen	21.3 ± 1.7	a	11.2 ± 1.1	ab	69.6 ± 6.8	a	102.1 ± 9.0	a	3.2 ± 0.4	a	2.5 ± 0.2	a	8.3 ± 0.9	a	13.9 ± 1.4	a	102.4 ± 5.4	a	116.3 ± 5.7	a
Barke	21.5 ± 3.3	a	10.3 ± 1.4	a	66.9 ± 6.6	a	98.8 ± 10.7	a	2.5 ± 0.3	a	2.5 ± 0.2	a	7.8 ± 0.5	a	12.9 ± 1.0	a	99.6 ± 9.7	a	112.4 ± 10.6	a
Baronesse	20.6 ± 0.4	a	13.4 ± 1.1	ab	79.4 ± 1.7	a	113.4 ± 2.7	a	3.0 ± 0.6	a	2.8 ± 0.1	a	8.3 ± 1.3	a	14.1 ± 1.9	a	107.5 ± 2.3	a	121.6 ± 3.2	a
Braemar	20.7 ± 3.9	a	14.6 ± 2.2	ab	81.9 ± 4.4	a	117.1 ± 6.4	a	2.7 ± 0.1	a	2.6 ± 0.3	a	9.4 ± 0.6	a	14.7 ± 1.0	a	118.9 ± 7.8	a	133.7 ± 7.8	a
Carina	30.0 ± 4.6	a	11.4 ± 1.1	ab	74.9 ± 7.4	a	116.2 ± 12.9	a	3.6 ± 0.5	a	3.1 ± 0.4	a	11.6 ± 1.6	a	18.3 ± 2.0	a	112.5 ± 8.5	a	130.7 ± 10.2	a
Grace	21.0 ± 2.3	a	10.4 ± 0.3	a	66.8 ± 3.5	a	98.1 ± 5.7	a	2.8 ± 0.3	a	2.4 ± 0.3	a	7.6 ± 1.1	a	12.8 ± 1.3	a	119.6 ± 6.8	a	132.4 ± 7.3	a
IPZ 24727	25.6 ± 5.0	a	17.9 ± 2.1	b	92.1 ± 6.1	a	135.6 ± 12.8	a	3.9 ± 0.5	a	3.4 ± 0.2	a	11.2 ± 1.0	a	18.5 ± 1.5	a	126.7 ± 12.6	a	145.2 ± 13.1	a
Irina	27.9 ± 2.2	a	10.4 ± 0.3	a	74.4 ± 5.5	a	112.7 ± 7.9	a	3.1 ± 0.3	a	2.4 ± 0.4	a	10.8 ± 1.0	a	16.3 ± 1.1	a	113.6 ± 4.1	a	129.9 ± 4.3	a
Mackay	21.2 ± 2.3	a	12.9 ± 1.8	ab	63.2 ± 8.7	a	97.3 ± 12.6	a	2.8 ± 0.6	a	2.3 ± 0.5	a	7.7 ± 1.1	a	12.8 ± 2.1	a	98.0 ± 7.2	a	110.8 ± 9.1	a
Marthe	25.8 ± 1.1	a	12.9 ± 1.0	ab	81.2 ± 3.2	a	119.8 ± 2.6	a	3.1 ± 0.1	a	3.0 ± 0.1	a	8.6 ± 0.5	a	14.6 ± 0.6	a	117.1 ± 3.4	a	131.7 ± 3.8	a
Melius	26.6 ± 2.6	a	12.2 ± 1.0	ab	79.6 ± 4.0	a	118.5 ± 6.8	a	3.0 ± 0.4	a	2.2 ± 0.6	a	8.7 ± 0.3	a	13.9 ± 1.2	a	112.3 ± 6.0	a	126.2 ± 7.2	a
Power	22.0 ± 2.0	a	9.3 ± 1.1	a	64.9 ± 4.5	a	96.2 ± 7.2	a	2.9 ± 0.4	a	2.0 ± 0.3	a	8.8 ± 1.4	a	13.8 ± 1.5	a	98.6 ± 8.4	a	112.4 ± 9.1	a
Quench	24.6 ± 4.6	a	12.3 ± 1.6	ab	74.3 ± 7.5	a	111.2 ± 13.4	a	2.9 ± 0.6	a	2.4 ± 0.2	a	9.2 ± 1.1	a	14.5 ± 1.9	a	105.0 ± 10.3	a	119.5 ± 12.0	a
Salome	19.7 ± 1.2	a	14.2 ± 1.4	ab	67.5 ± 2.4	a	101.4 ± 4.9	a	2.2 ± 0.2	a	2.4 ± 0.2	a	7.4 ± 1.6	a	11.9 ± 2.0	a	100.3 ± 4.6	a	112.2 ± 6.4	a
Scarlett	21.8 ± 2.9	a	13.0 ± 0.8	ab	73.3 ± 3.2	a	108.1 ± 6.3	a	2.1 ± 0.3	a	2.6 ± 0.4	a	7.7 ± 1.0	a	12.4 ± 1.7	a	122.8 ± 0.5	a	135.2 ± 1.8	a
Shakira	27.0 ± 3.2	a	10.3 ± 1.6	a	83.2 ± 3.8	a	120.5 ± 8.0	a	3.9 ± 0.6	a	2.5 ± 0.5	a	10.1 ± 1.0	a	16.5 ± 1.8	a	107.0 ± 6.4	a	123.4 ± 8.1	a
Sissy	29.6 ± 6.5	a	11.6 ± 2.2	ab	69.3 ± 6.6	a	110.4 ± 14.5	a	3.1 ± 0.5	a	2.6 ± 0.4	a	9.9 ± 0.5	a	15.5 ± 1.1	a	114.8 ± 15.0	a	130.3 ± 16.1	a
Solist	25.0 ± 0.9	a	12.2 ± 0.4	ab	72.0 ± 2.0	a	109.2 ± 2.5	a	2.7 ± 0.3	a	2.7 ± 0.2	a	9.6 ± 0.0	a	15.0 ± 0.3	a	109.5 ± 9.1	a	124.5 ± 9.4	a
Trumpf	26.1 ± 0.6	a	10.8 ± 0.3	a	69.9 ± 1.6	a	106.7 ± 1.5	a	3.1 ± 0.1	a	2.4 ± 0.2	a	9.5 ± 0.6	a	15.0 ± 0.9	a	100.3 ± 3.4	a	115.3 ± 4.1	a
Union	27.0 ± 1.5	a	15.4 ± 0.8	ab	75.1 ± 2.9	a	117.5 ± 3.0	a	2.8 ± 0.3	a	2.7 ± 0.1	a	8.8 ± 0.3	a	14.3 ± 0.6	a	114.4 ± 4.7	a	128.7 ± 5.1	a
Ursa	22.4 ± 1.8	a	11.2 ± 0.2	ab	78.7 ± 3.6	a	112.3 ± 5.4	a	2.7 ± 0.3	a	2.4 ± 0.5	a	10.1 ± 0.3	a	15.2 ± 0.7	a	107.9 ± 7.9	a	123.1 ± 7.9	a
Volla	24.0 ± 2.0	a	11.6 ± 1.1	ab	76.4 ± 4.2	a	112.0 ± 6.7	a	3.0 ± 0.2	a	2.9 ± 0.1	a	10.0 ± 0.3	a	15.9 ± 0.5	a	114.2 ± 5.1	a	130.1 ± 5.6	a
Wiebke	23.6 ± 4.2	a	11.4 ± 2.3	ab	67.6 ± 4.7	a	102.5 ± 11.0	a	2.4 ± 0.3	a	1.8 ± 0.4	a	9.2 ± 1.4	a	13.4 ± 1.4	a	106.9 ± 10.8	a	120.3 ± 12.2	a
**Mean**	24.1		12.2		74.0		110.3		2.9		2.5		9.1		14.6		110.0		124.6	
**Min**	19.7		9.3		63.2		96.2		2.1		1.8		7.4		11.9		98.0		110.8	
**Max**	30.0		17.9		92.1		135.6		3.9		3.4		11.6		18.5		126.7		145.2	

Supplementary Table 8 Mean (± SE) and Tukey-test for nitrogen (N) translocation of leaves, sheaths, (stems+ears) and shoot total from pre-anthesis to grain weight at maturity, the grain N uptake from post-anthesis, and total N translocation efficiency (NTE) in 2015. Mean comparisons among varieties from Tukey´s HSD test indicate a significant difference at $p < 0.05$. The same letters mean groups in a column that were not significantly different from one another.

Variety Name	Translocation (kg ha^{-1})								Post-anthesis N uptake (kg ha^{-1})		NTE (%)		Contribution			
	Leaves		Sheaths		Stems+ears		Shoot total						Assimilation pre-anthesis		Assimilation post-anthesis	
	mean±SE		mean±SE		mean±SE		mean±SE		mean±SE		mean±SE		mean±SE		mean±SE	
Aspen	18.1 ± 1.6	a	8.8 ± 0.9	ab	61.4 ± 6.7	ab	88.3 ± 8.5	a	14.1 ± 5.0	ab	86.2 ± 1.4	a	85.8 ± 5.7	ab	14.2 ± 0.9	ab
Barke	19.0 ± 3.0	a	7.8 ± 1.3	a	59.1 ± 6.1	ab	85.9 ± 9.9	a	13.7 ± 5.2	ab	86.7 ± 0.9	a	86.1 ± 5.0	ab	13.9 ± 0.8	ab
Baronesse	17.6 ± 0.6	a	10.6 ± 1.0	ab	71.1 ± 1.8	ab	99.3 ± 2.1	a	8.2 ± 1.3	a	87.6 ± 1.5	a	92.4 ± 1.1	ab	7.6 ± 0.1	ab
Braemar	18.0 ± 3.8	a	12.0 ± 2.1	ab	72.4 ± 4.2	ab	102.4 ± 5.9	a	16.5 ± 5.8	ab	87.4 ± 0.8	a	86.6 ± 4.1	ab	13.4 ± 0.6	ab
Carina	26.4 ± 4.3	a	8.3 ± 0.7	ab	63.3 ± 6.1	ab	98.0 ± 11.0	a	14.5 ± 3.1	ab	84.2 ± 0.5	a	86.3 ± 4.2	ab	13.7 ± 0.7	ab
Grace	18.2 ± 2.1	a	7.9 ± 0.5	a	59.2 ± 3.2	ab	85.3 ± 4.7	a	34.3 ± 3.9	b	87.0 ± 0.7	a	71.4 ± 2.1	a	28.6 ± 0.9	a
IPZ 24727	21.7 ± 4.7	a	14.5 ± 1.9	b	80.9 ± 6.1	b	117.0 ± 12.2	a	9.7 ± 1.4	a	86.1 ± 1.1	a	92.3 ± 1.2	ab	7.7 ± 0.1	ab
Irina	24.8 ± 2.0	a	8.0 ± 0.1	a	63.6 ± 5.8	ab	96.4 ± 7.6	a	17.2 ± 4.9	ab	85.4 ± 1.3	a	84.6 ± 4.7	ab	15.4 ± 0.9	ab
Mackay	18.3 ± 1.9	a	10.6 ± 1.3	ab	55.5 ± 8.0	a	84.5 ± 10.9	a	13.5 ± 6.8	ab	86.8 ± 1.1	a	85.7 ± 8.0	ab	14.3 ± 1.3	ab
Marthe	22.7 ± 1.0	a	9.9 ± 0.9	ab	72.6 ± 3.3	ab	105.2 ± 2.6	a	11.9 ± 5.2	ab	87.8 ± 0.5	a	90.2 ± 4.2	ab	9.8 ± 0.5	ab
Melius	23.6 ± 2.2	a	10.0 ± 0.7	ab	70.9 ± 3.6	ab	104.6 ± 5.6	a	7.7 ± 2.5	a	88.4 ± 0.5	a	93.2 ± 2.1	ab	6.8 ± 0.2	ab
Power	19.1 ± 1.7	a	7.3 ± 1.3	a	56.1 ± 3.5	a	82.5 ± 6.0	a	16.1 ± 6.3	ab	85.7 ± 0.9	a	84.3 ± 5.2	ab	15.7 ± 1.0	ab
Quench	21.6 ± 4.2	a	10.0 ± 1.5	ab	65.1 ± 6.5	ab	96.7 ± 11.8	a	8.3 ± 1.6	a	86.9 ± 0.7	a	91.4 ± 2.3	b	8.6 ± 0.2	b
Salome	17.5 ± 1.0	a	11.8 ± 1.2	ab	60.1 ± 0.8	ab	89.5 ± 2.9	a	10.7 ± 3.0	ab	88.4 ± 1.3	a	89.6 ± 2.9	ab	10.4 ± 0.3	ab
Scarlett	19.7 ± 2.7	a	10.4 ± 0.6	ab	65.6 ± 2.6	ab	95.7 ± 5.0	a	27.1 ± 4.9	ab	88.7 ± 1.2	a	77.9 ± 4.0	ab	22.1 ± 1.1	ab
Shakira	23.1 ± 2.7	a	7.8 ± 1.2	a	73.1 ± 3.0	ab	104.0 ± 6.2	a	2.9 ± 0.6	a	86.4 ± 0.7	a	97.3 ± 0.5	b	2.7 ± 0.0	b
Sissy	26.5 ± 6.1	a	9.0 ± 2.0	ab	59.4 ± 6.3	ab	94.8 ± 13.5	a	19.9 ± 5.5	ab	85.5 ± 1.0	a	82.5 ± 4.0	ab	17.5 ± 0.8	ab
Solist	22.3 ± 0.6	a	9.5 ± 0.4	ab	62.4 ± 1.9	ab	94.2 ± 2.3	a	15.3 ± 6.9	ab	86.2 ± 0.2	a	87.3 ± 5.3	ab	12.7 ± 0.8	ab
Trumpf	23.0 ± 0.5	a	8.3 ± 0.3	ab	60.4 ± 1.8	ab	91.7 ± 1.2	a	8.6 ± 4.2	a	86.0 ± 0.7	a	91.8 ± 3.8	ab	8.2 ± 0.3	ab
Union	24.2 ± 1.3	a	12.7 ± 0.9	ab	66.3 ± 2.8	ab	103.2 ± 2.4	a	11.2 ± 3.0	ab	87.9 ± 0.3	a	90.5 ± 2.4	ab	9.5 ± 0.3	ab
Ursa	19.7 ± 1.8	a	8.8 ± 0.5	ab	68.6 ± 4.0	ab	97.1 ± 5.7	a	10.8 ± 5.2	ab	86.4 ± 1.1	a	90.6 ± 4.3	ab	9.4 ± 0.4	ab
Volla	21.0 ± 1.9	a	8.7 ± 1.0	ab	66.4 ± 4.2	ab	96.1 ± 6.4	a	18.1 ± 4.6	ab	85.7 ± 0.7	a	84.2 ± 4.0	ab	15.8 ± 0.8	ab
Wiebke	21.2 ± 3.9	a	9.6 ± 2.3	ab	58.3 ± 3.7	ab	89.1 ± 9.8	a	17.8 ± 5.8	ab	86.9 ± 0.6	a	83.5 ± 4.2	ab	16.5 ± 0.8	ab
**Mean**	21.2		9.7		64.9		95.7		14.3		86.7		87.2		12.8	
**Min**	17.5		7.3		55.5		82.5		2.9		84.2		71.4		2.7	
**Max**	26.5		14.5		80.9		117.0		34.3		88.7		97.3		28.6	
